# Genome-directed analysis of prophage excision, host defence systems, and central fermentative metabolism in *Clostridium pasteurianum*

**DOI:** 10.1038/srep26228

**Published:** 2016-09-19

**Authors:** Michael E. Pyne, Xuejia Liu, Murray Moo-Young, Duane A. Chung, C. Perry Chou

**Affiliations:** 1 Department of Chemical Engineering, University of Waterloo, Waterloo, Ontario, Canada; 2 Department of Pathology and Molecular Medicine, McMaster University, Ontario, Canada; 3 Algaeneers Inc. and Neemo Inc., Hamilton, Ontario, Canada

## Abstract

*Clostridium pasteurianum* is emerging as a prospective host for the production of biofuels and chemicals, and has recently been shown to directly consume electric current. Despite this growing biotechnological appeal, the organism’s genetics and central metabolism remain poorly understood. Here we present a concurrent genome sequence for the *C. pasteurianum* type strain and provide extensive genomic analysis of the organism’s defence mechanisms and central fermentative metabolism. Next generation genome sequencing produced reads corresponding to spontaneous excision of a novel phage, designated φ6013, which could be induced using mitomycin C and detected using PCR and transmission electron microscopy. Methylome analysis of sequencing reads provided a near-complete glimpse into the organism’s restriction-modification systems. We also unveiled the chief *C. pasteurianum* Clustered Regularly Interspaced Short Palindromic Repeats (CRISPR) locus, which was found to exemplify a Type I-B system. Finally, we show that *C. pasteurianum* possesses a highly complex fermentative metabolism whereby the metabolic pathways enlisted by the cell is governed by the degree of reductance of the substrate. Four distinct fermentation profiles, ranging from exclusively acidogenic to predominantly alcohologenic, were observed through redox consideration of the substrate. A detailed discussion of the organism’s central metabolism within the context of metabolic engineering is provided.

*Clostridium pasteurianum* is an obligately anaerobic, endospore-forming soil bacterium that is emerging as an attractive industrial host owing to its unique fermentative metabolism^1^,^2^,^3^ and newfound capacity to directly consume electric current^4^. Growth of *C. pasteurianum* on conventional sugars, such as glucose or sucrose, yields a butyric acid fermentation characteristic of the clostridia^5^,^6^. Conversely, growth on glycerol leads to a marked shift in metabolism distinguished by an alcohologenic profile comprised of 1,3-propanediol and butanol^5^,^7^,^8^. The glycerol fermentation carried out by *C. pasteurianum* has drawn significant attention to the organism in light of the recent growth in global biodiesel production, which has generated an abundance of crude glycerol, now considered a waste-stream, rather than a valued co-product^9^,^10^,^11^,^12^. Crude glycerol is present at approximately 10% (w/w) of the final biodiesel preparation, causing its value to sharply decline in accordance with expansion of the biodiesel industry. Consequently, abundant and inexpensive waste glycerol has found application in various processes, including animal-feeding, composting, anaerobic digestion, and a range of other thermochemical and biological conversions^12^,^13^. Owing to the vast metabolic diversity found in nature, the biotechnological route of waste glycerol valorization is often regarded as the most promising^10^. Fermentation of glycerol, naturally carried out by species of *Klebsiella*, *Citrobacter*, and *Clostridium*^14^, in addition to engineered *E. coli*^15^, offers an array of value-added bioproducts, including ethanol, butanol, 1,2- and 1,3-propanediol, and 2,3-butanediol. While propanediol and butanediol are important chemical building blocks, butanol serves as a prospective biofuel that is superior to ethanol in both physicochemical and fuel properties^16^. In nature, *C. pasteurianum* is the only organism known to convert glycerol as a sole carbon and energy source into butanol^1^. Despite its promise, however, the *C. pasteurianum* glycerol fermentation is currently one of the most poorly understood glycerol-to-biofuel processes.

Bacteria defend against bacteriophages (phages), plasmids, and other invading nucleic acids through the use of primitive cellular immune systems. The chief prokaryotic defence mechanisms are restriction-methylation (RM) and Clustered Regularly Interspaced Short Palindromic Repeats (CRISPR) systems, both of which utilize endonuclease-mediated attack and afford host cells with self versus non-self discrimination^17^,^18^. Restriction endonucleases target DNAs for attack through recognition of short, typically palindromic, recognition sequences, whereby self-restriction is blocked by host methylation^18^. On the other hand, CRISPR systems provide adaptive immunity through the use of host-encoded DNA sequence tags specific to an invading element^19^. These sequence tags, or spacers, are flanked by short partially palindromic repeats (24–47 bp) and provide the basis for immunity against future invasions^19^. CRISPR arrays are dynamic in nature, as new spacers are rapidly acquired in response to predation and unused spacers are excised from the host genome. CRISPR-associated (Cas) proteins, involved in both acquisition of new spacer sequences and subsequent attack of invading elements, are often found in close proximity to CRISPR arrays within bacterial and archaeal genomes^20^. Gene mining tools and comprehensive online databases, such as REBASE^21^ and CRISRdb^22^, enable simple identification of putative RM and CRISPR systems within bacterial genomes. Next generation sequencing also offers an avenue for discovery of novel RM systems through sensitive detection of host-modified nucleotides^23^. As phage attack is often implicated as a key factor in the historic failure of large-scale clostridial acetone and butanol (AB) production^24^, identification and characterization of clostridial defence systems could provide a means of engineering immunity against phage predation and associated culture deterioration^25^. In this context, genomic analysis provides an opportunity to assess phage content of bacterial genomes^26^, including the presence of intact and active prophages^27^, which constitutes an important yet largely unexamined facet of clostridial biology.

A small number of studies have investigated the potential of *C. pasteurianum* to produce butanol from crude glycerol^2^,^3^,^28^,^29^,^30^. To complement such efforts, techniques have recently been developed allowing high level electrotransformation and chromosomal gene disruption and deletion using *C. pasteurianum*^31^,^32^,^33^, thus paving the way for rational metabolic engineering and strain optimization efforts^34^. A full genome sequence is available for an environmental isolate of *C. pasteurianum* (strain BC1) (unpublished data) and two completed genome sequences have recently been announced for the type strain [ATCC 6013 (DSM 525)]^35^,^36^. In addition to an expanding repertoire of genetic tools and genome sequencing data, it is clear that a better understanding of the central fermentative pathways of *C. pasteurianum* is paramount to the advancement of this organism for biotechnological valorization of crude glycerol^2^. In this study, we present a concurrent genome assembly for *C. pasteurianum* type strain ATCC 6013 (DSM 525) and provide detailed analysis of the organism’s unique fermentative metabolism. We show that the organism exhibits a highly flexible, branched central metabolism, where product distribution varies considerably between carbon sources and is dictated chiefly by redox characteristics of the fermentation substrate. To stimulate a more thorough understanding of *C. pasteurianum* genetics and general biology, we also provide insight into the organism’s defence mechanisms through analysis of the restriction-modification methylome and identification of a chief Type I-B CRISPR system. Finally, we provide evidence that the genome of *C. pasteurianum* encodes an intact prophage that is spontaneously excised under standard growth conditions and induced using mitomycin C. The detailed genomic analysis of *C. pasteurianum* presented herein will contribute to our understanding of substrate utilization and biofuel production by this promising organism, as well as provide a genetic and metabolic framework for rational strain engineering.

## Results and Discussion

### *C. pasteurianum* ATCC 6013 (DSM 525) genome closing and detection of an extrachromosomal circular bacteriophage excision product

**Figure 1:**
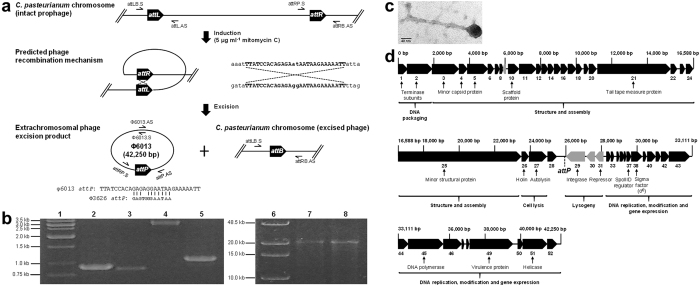
Identification and excision of phage φ6013 from the genome of *C. pasteurianum*. (**a**) Predicted excision mechanism of phage φ6013 from the genome of *C. pasteurianum*. Phage excision was induced by exposing exponential phase cultures of *C. pasteurianum* to 5 μg ml^−1^ mitomycin C, leading to recombination between *attL* and *attR* sites. Sequences corresponding to the core *attL* and *attR* φ6013 recombination sites are shown in uppercase. The resulting *attP* sequence of phage φ6013 is compared to the similar 12 nt core *attP* site of phage φ3626 from *C. perfringens*. Prophage excision leads to a circular 42,250 bp phage genome and a single *attB* scar site within the genome of *C. pasteurianum*. PCR primers for screening *attL*, *attR*, *attP*, and *attB* recombination sites are shown, as well as screening primers for long range PCR of the circular excised φ6013 genome. Genomes, genomic regions, and PCR primers are not depicted to scale. (**b**) PCR verification of phage φ6013 excision from the *C. pasteurianum* chromosome. Orientation and arrangement of PCR primers are depicted in Fig. 1a. Lane 1: marker; lane 2: 904 bp *attL* product (attLB.S + attL.AS); lane 3: 872 bp *attR* product (attRP.S + attRB.AS); lane 4: 3,154 bp *attP* product (attRP.S + attP.AS); lane 5: 1,076 bp *attB* product (attLB.S + attRB.AS); lane 6: long range PCR marker; lane 7: 22,756 bp 5′ φ6013 product (φ6013.S + attP.AS); lane 8: 22,678 bp 3′ φ6013 product (attRP.S + φ6013.AS). (**c**) Transmission electron microscopy image of phage φ6013 visualized at 245,000× magnification. (**d**) Genomic arrangement of phage φ6013 (42,250 bp). All 52 predicted genes, including some functional assignments, are depicted and are numbered consecutively. Genes in black and grey depict different directions of transcription. The predicted phage attachment site (*attP*) described in the main text is shown. All genes and intergenic regions are depicted to scale.

We recently reported a draft genome sequence of *C. pasteurianum* comprised of 12 contigs^37^. To join contigs, we employed an additional round of SMRT sequencing using a size-selected large insert library and the RS II analyzer (Pacific Biosciences; Menlo Park, CA), resulting in a draft genome sequence comprised of two contigs of sizes 4.37 Mbp (contig 1) and 13.2 kb (contig 2). Contig 2 was analyzed and found to be comprised of two regions (approximately 6.2 kb and 7.0 kb) that were also identified within contig 1. These regions possess 28 bp of overlap within contig 2, yet are separated by approximately 29.0 kb in contig 1 (see Supplementary Fig. S1). We analyzed the 29.0 kb intervening sequence between the 6.2 kb and 7.0 kb regions of contig 1 and identified a number of genes encoding putative phage and prophage gene products. PHAST analysis^26^ of the *C. pasteurianum* genome and a concurrent genome sequencing effort^33^ predicted an intact prophage within this region. Hence, we hypothesized that genome sequencing reads corresponding to contig 2 could have arisen from spontaneous excision of a circular, extrachromosomal phage or phage-like product from the genome of *C. pasteurianum*. It is possible that contig 2 (13.2 kb) corresponds to sequence generated directly from the excised phage (i.e. the phage genome) or phage-like product. Further, the 28 bp overlap sequence between the 6.2 kb and 7.0 kb regions in contig 2 was found to be preserved within the 5′ terminus of the 6.2 kb region and the 3′ terminus of the 7.0 kb region of contig 1 (see Supplementary Fig. S1). This sequence arrangement is consistent with a chromosomal excision event, where the 28 bp overlap sequence of contig 2 represents the phage attachment site following excision (*attP*) and the corresponding 28 bp sites within the 6.2 kb and 7.0 kb regions of contig 1 are the respective left (*attL*) and right (*attR*) phage attachment sites within the chromosome of *C. pasteurianum* (i.e. prior to phage excision) (Fig. 1a). Chromosomal phage integration presumably occurred through homology between the free phage *attP* site and the bacterial chromosome (*attB*), whereas subsequent excision of the prophage proceeded via recombination between *attL* and *attR*. Based on this hypothesis, phage excision should result in a single chromosomal “scar” site (*attB*) within the phage-less *C. pasteurianum* genome. We confirmed this hypothesis by successfully amplifying the phage-less bacterial chromosome region (*attB*), as well as products corresponding to the unexcised prophage (*attL* and *attR*) and the excised circular phage product (*attP*) (Fig. 1b). All four PCR products were of the expected sizes based on the proposed phage excision event and Sanger DNA sequencing revealed the expected nucleotide sequences (data not shown). In the absence of excision, the *attB* PCR primer set is expected to generate a 43.3 kb product, which is beyond the amplification limits of PCR. We were also successful in PCR-amplifying overlapping 22.7 kb and 22.8 kb products, which both span the *attP* attachment site and together comprise the full-length excised phage genome (Fig. 1a,b). Furthermore, the orientation of primers utilized to generate these products confirms circularity of the excised phage product.

Although activation of the *C. pasteurianum* prophage occurred spontaneously in the aforementioned analysis, prophage excision can be artificially induced using ultraviolet irradiation or mitomycin C, a potent antibiotic^38^,^39^. To assess phage induction, we exposed growing cultures of *C. pasteurianum* to varying concentrations of mitomycin C (0, 1, 2.5, 5 and 10 μg ml^−1^) and monitored culture turbidity for signs of phage release and cell lysis, characterized by a dramatic decline in OD_600_^27^. The culture containing 5 μg ml^−1^ mitomycin C exhibited a dramatic decrease in OD_600_ approximately four hours following induction. Imaging via TEM of the 5.0 μg ml^−1^ mitomycin C phage lysates revealed an abundance of phages with long tails possessing short terminal fibers (Fig. 1c). We measured tail size of seven distinct, well-resolved phages, resulting in an average length of 242 ± 11 nm. Based on these observations, we hypothesize that the excised phage, which we designate φ6013, belongs to the *Siphoviridae* family of bacteriophages^27^. The excised φ6013 phage possesses a 42,250 bp circular genome with a GC content of 33.2%, which is greater than the GC content of the *C. pasteurianum* chromosome (29.9%). A total of 52 protein-coding genes, possessing homology to both phage and bacterial genes, are annotated in the phage genome, 48 of which are transcribed in the same direction (Fig. 1d; see Supplementary Table S1). Phage gene products can be grouped into packaging and structural proteins (capsid, tail, terminase, and portal proteins), lysis proteins (holin and endolysin), lysogeny proteins (integrase and repressor), and DNA modification, replication, and gene expression proteins (DNA polymerase and helicase). Since proteins for both cell lysis (a holin and autolysin) and lysogeny (an integrase and cI-like transcriptional repressor) could be identified, φ6013 clearly embodies a temperate phage. PHAST analysis^26^ of φ6013 gene products revealed a total of 39 proteins possessing significant protein identity to other phages. Of particular relevance are the *C. perfringens* φCP51^38^ (18 protein matches) and φ3626 phages^39^ (5 protein matches), both of which are part of the *Siphoviridae* family. Interestingly, φ6013, in addition to φCP51 and φ3626^38^,^39^, encodes a sporulation-specific transcriptional regulator (*spoIIID*) and RNA polymerase sigma factor (*sigE*), indicating a potential relationship between φ6013 and sporulation of *C. pasteurianum*. Spore titers have been reported to differ between seemingly identical strains of *C. pasteurianum* obtained from different culture collections (ATCC and DSM)^36^, which could reflect variation in levels of excision of φ6013. Genome sequences previously reported for the ATCC and DSM type strains both contain the intact φ6013 prophage, as it is presumed that the authors did not generate sequencing reads corresponding to the excised phage. Since we observed relatively high-levels of spontaneous phage excision in this study, corresponding to a sequencing coverage of 35–60×, it would be advantageous to determine which set of conditions, if any, are responsible for activation of phage φ6013.

Following elucidation of the φ6013 excision mechanism, a single unclosed 4.37 Mbp contig remained (i.e. contig 1), which was found to be approximately 22 kb larger than previously reported *C. pasteurianum* genome sequences^35^,^36^. Based on extensive long range PCR analyses, we provide evidence that the *C. pasteurianum* contig gap is part of a large 93.5 kb chromosomal duplication (see Supplementary Note and Supplementary Fig. S2). This duplication, which is flanked by genes encoding transposable elements, is not present in previous *C. pasteurianum* genome sequences and its large size prevented closing of our draft assembly using traditional PCR methods. Hence, our current genome assembly is comprised of a single unclosed contig.

### General *C. pasteurianum* genome characteristics

**Figure 2:**
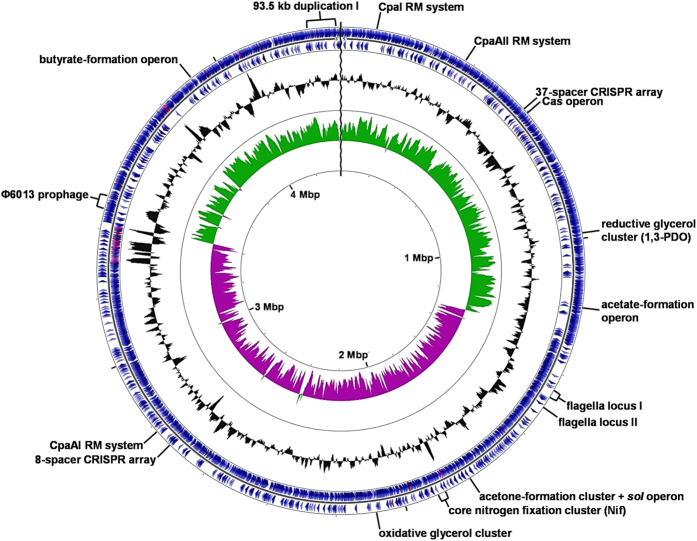
The chromosome of *C. pasteurianum* ATCC 6013. Contig 1 (4,373,654 bp) is depicted as a circular chromosome and shows the approximate location of key genomic features discussed in this study. The two outermost circles indicate locations of gene coding regions (blue) in plus (circle one) and minus (circle two) strands. Genes encoding tRNAs and rRNAs are shown in fuchsia and lavender, respectively. Circle three shows G + C content (deviation from average) and circle four depicts G + C skew in plus (green) and minus (purple) strands. Genome scale is indicated in Mbp on the innermost circle. The CGView Server^120^ was used to construct the genome map.

Based on the hypothesis outlined above, the expected size of the *C. pasteurianum* genome is 4,444,510 bp. Our current draft genome is comprised of a single 4,373,654 bp contig possessing a GC content of 29.9% (Fig. 2). No plasmids could be identified. The size of the genome is within the range of most clostridia (3–5 Mbp^34^). Based on 16S rRNA phylogeny, *C. pasteurianum* is most closely related to (from most related to least related) *C. acidisoli*, *C. akagii*, *C. arbusti*, and *C. carboxidivorans*^40^, yet complete genome sequences are not available for these species. *C. acetobutylicum*, *C. botulinum*, *C. autoethanogenum*, and *C. ljundahlii* are the closest relatives with fully-sequenced genomes (see Supplementary Table S2)^40^. Currently six *C. pasteurianum* genome sequencing projects are underway or have been completed^35^,^36^,^37^,^41^,^42^, highlighting the recent emergence of *C. pasteurianum* as a promising industrial producer of butanol. These genome efforts encompass three finished genome sequences and three draft sequences, collectively covering three distinct strains of the species. As outlined above, one concurrent effort recently reported two closed genome sequences of the *C. pasteurianum* type strain from two culture collections (ATCC 6013 and DSM 525)^35^,^36^. A brief comparison of the genome sequence reported in this study with other *C. pasteurianum* sequencing projects is provided in Table 1. The *C. pasteurianum* genome is predicted to possess 3,803 protein coding genes, 81 tRNA genes, and 30 rRNA genes (Fig. 2). Approximately 75% of genes in the genome could be assigned a function based on Cluster of Orthologous Groups (COGs), while 1,006 genes (approximately 25% of genes) were annotated as general function only (COG function R) or function unknown (COG function S). Aside from poorly characterized genes, the largest COGs, collectively comprising approximately one third of all protein-coding genes (32.3%), were ones involved in amino acid transport/metabolism (COG function E; 12% of genes), energy production/conversion (COG function C; 10% of genes), and carbohydrate transport/metabolism (COG function G; 10% of genes). The large proportion of genes involved in metabolism and energy production is testament to the exceptional metabolic flexibility exhibited by *C. pasteurianum*.

*C. pasteurianum* is a flagellated bacterium and two large (approximately 25.7 kb and 14.5 kb) flagella loci were identified in the genome and together encode all core flagellar structural genes, including genes involved in filament [*fliC* (CP6013_1370, CP6013_1401)], hook [*flgE* (CP6013_1358)], and rod [*flgB* (CP6013_1369), *flgC* (CP6013_1368), and *flgG* (CP6013_1343, CP6013_1344)] formation. A collection of chemotaxis genes are located immediately downstream of the flagellar loci, while *motA* (CP6013_3023) and *motB* (CP6013_3022) chemotaxis genes are encoded at a distant location in the genome. *C. pasteurianum* is believed to be the first isolated nitrogen-fixing organism and nitrogen fixation from cell-free lysates was first observed using *C. pasteurianum*^43^. Cell-free extracts convert atmospheric N_2_ into ammonia using ferredoxin as electron donor and ATP to drive the reaction^44^. A core cluster of key nitrogen fixation genes (*nif*), including genes encoding the MoFe dinitrogenase (*nifDK*; CP6013_1738 and CP6013_1737) and Fe dinitrogenase reductase (*nifH1*; CP6013_1739) protein components^45^, is present within a 29.9 kb region of the genome (CP6013_1731–1754) and has been described extensively in *C. pasteurianum*^46^ and other nitrogen-fixers^47^. This region also encodes *nifE* (CP6013_1736), *nifN-B* (CP6013_1735), *nifC* (CP6013_1733), *nifV1* (CP6013_1731), and *nifV2* (CP6013_1732) genes involved in nitrogenase assembly and Mo/Fe insertion. It has been reported that *C. pasteurianum* possesses a total of six *nifH* and *nifH*-like genes^48^,^49^, which we confirmed with BLAST analysis using NifH1 as a protein query. Four of the five NifH-like amino acid sequences [NifH2 (CP6013_1740), NifH4 (CP6013_3825), NifH5 (CP6013_2037), and NifH6 (CP6013_1749)] possess substantial sequence identity to NifH1 (92–99%), while NifH3 (CP6013_3385) was found to contain 64% of NifH1 amino acid identities. NifH5 (CP6013_2037) was not found to be associated with other nitrogenase components, while NifH4 (CP6013_3825) was identified within a smaller nitrogen-fixing cluster (CP6013_3825–3832) containing putative *nifK* (CP6013_3827) and *nifE* (CP6013_3826 and CP6013_3832) genes, as well as two *nifB* or *nifB*-like genes (CP6013_3829 and CP6013_3830). Interestingly, three *nifH*-like genes, *nifH5* (CP6013_2037), *nifH3* (CP6013_3385), and nifH4 (CP6013_3825), are found adjacent to genes encoding putative transposases (CP6013_2035, CP6013_3386, and CP6013_3824, respectively). NifH3 (CP6013_3385) is the only NifH-like protein that is not transcribed under nitrogen fixation conditions, as it exemplifies a Mo-independent Fe-nitrogenase^50^. Genes encoding the Fe-nitrogenase component (*anfDGK*; CP6013_3387–3389) are close to the Fe dinitrogenase reductase gene (*nifH3*/*anfH*; CP6013_3385). In addition to Mo-dependent (Nif) and Mo-independent (Anf) nitrogenases, it has been reported that *C. pasteurianum* harbors a vanadium-dependent nitrogen-fixing (Vnf) system^51^. We identified a putative vanadium-dependent nitrogenase locus, represented by *vnfD* (CP6013_1748), *vnfG* (CP6013_1747), and *vnfK* (CP6013_1746), which are positioned adjacent to *nifH6* (CP6013_1749).

### Analysis of the *C. pasteurianum* methylome and restriction-modification systems

To gain further insight into the cellular defence mechanisms of *C. pasteurianum* and guide future genetic work with this organism, we analyzed the organism’s methylome using SMRT sequencing data^37^. Only m6A modifications could be detected in this study, as sequence coverage was insufficient for m5C detection. Methylome analysis unveiled four distinct m6A methylation motifs (Table 2). Two such motifs are associated with experimentally-verified restriction activities. The first is CpaI^18^, a Type II system with a recognition sequence of 5′-GATC-3′ that is common within the *Clostridium* genus^52^. We previously identified and characterized restriction activity corresponding to the other m6A system detected using methylome analysis^32^. This Type I restriction-methylation-sensitivity (RMS) system, with a predicted recognition sequence of 5′-AAGNNNNNCTCC-3′ (N = any nucleotide; A, C, G, or T), was designated CpaAII. Interestingly, only 37.8% of CpaI recognition sites within the genome were found to be methylated, while 92.9% of CpaAII sites were modified. Two new m6A-specific methylation motifs were also detected in this study, with recognition sequences of 5′-GRTAAAG-3′ and 5′-CAAAAAR-3′ (R = purine; A or G). In addition to the m6A-specific RM activities outlined above, a third experimentally-verified RM system has been elucidated in the type strain of *C. pasteurianum*. This system, designated CpaAI (5′-CGCG-3′)^52^, is m5C-specific, and thus was not detected in this study.

Based on analysis by REBASE^21^, the genome of *C. pasteurianum* is predicted to encode a total of eight methyltransferase genes, which ostensibly includes the single Type I RMS system, two Type II RM systems, a single Type II protein with dual R + M activities, and two lone Type II M proteins lacking associated R activity. The CpaAII Type I RMS system (5′-AAGNNNNNCTCC-3′) is encoded by genes CP6013_0336, CP6013_0337, and CP6013_0338, respectively, while genes CP6013_2557 and CP6013_2558 represent the CpaAI (5′-CGCG-3′) Type II RM proteins, respectively. Whereas gene CP6013_0098 corresponds to the R protein of the Type II CpaI system (5′-GATC-3′), three consecutive adjacent genes (CP6013_0095–0097) could putatively encode the associated M activity. Based on REBASE^21^ and our methylome data, it is predicted that gene CP6013_1459 encodes a lone M protein with a recognition sequence of 5′-CAAAAAR-3′, gene CP6013_0727 encodes the dual RM protein possessing 5′-GRTAAAG-3′ recognition, and gene CP6013_0738 codes for the remaining lone M protein, with an unknown recognition sequence. Note that methylation by the CP6013_0738-encoded methyltransferase was undetected in this study though the gene shares similarity with a silent non-specific methyltransferase gene often found within prophages. Such prophage genes can only be activated upon cloning into a plasmid. Finally, it is noteworthy that *C. pasteurianum* appears to restrict DNA substrates possessing CpG (5′-CG-3′) or GpC (5′-GC-3′) methylation based on plasmid transformation assays^31^, suggesting the presence of a methylation-dependent restriction endonuclease in *C. pasteurianum*. While wild-type *E. coli* cleaves DNA substrates containing m5C^53^, Type IV methylation-dependent restriction endonucleases are widespread in bacteria^21^. However, the genetic basis corresponding to the putative methylation-dependent restriction activity observed in *C. pasteurianum* could not be deciphered based on the genomic analysis performed in this study.

### Identification of putative CRISPR systems

**Figure 3:**
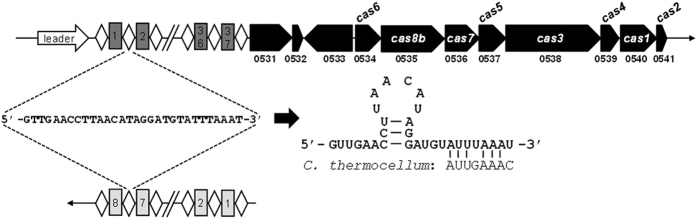
Genomic analysis of the central Type I-B CRISPR system of *C. pasteurianum*. Structure and orientation of CRISPR arrays and *cas* genes within the genome of *C. pasteurianum* are shown. Numbers below genes specify locus tags (CP6013 prefix is omitted). Three genes, encoding a putative histidine kinase (CP6013_0531), transposase (CP6013_0532), and a hypothetical protein (CP6013_0533), are located between the 37-spacer CRISPR array and *cas* genes. Genes encoding the Type I-B Cas proteins are located adjacent to a 37-spacer CRISPR array (spacers are depicted as dark gray boxes). A second 8-spacer CRISPR array (spacers are depicted as light gray boxes) possessing the same 30 nt direct repeat sequence (diamonds) was found elsewhere in the *C. pasteurianum* chromosome, separated from the *cas* genes by approximately 2.1 Mbp. The sequence of the common 30 nt direct repeat sequence is shown corresponding to the direction of transcription, which is in opposite directions. A predicted RNA folded structure of the 30 nt direct repeat is shown and compared to the 8 nt 5′ tag of mature crRNA from the *C. thermocellum* Type I-B system. A putative leader sequence is depicted upstream of the 37-spacer array, while the presence of a similar element within the 8-spacer array is not clear.

Approximately 45% of bacterial genomes encode CRISPR-associated (Cas) proteins and putative CRISPR arrays comprised of repetitive repeat-spacer units^22^. Surprisingly, 20 of 27 (74%) clostridial genomes are predicted to encode CRISPR-Cas systems^22^,^25^, compared to only 44% of Firmicutes^54^. Hence, we analyzed the genome of *C. pasteurianum* for putative CRISPR arrays and Cas-encoding genes using CRISPRfinder^55^. Two putative CRISPR arrays were identified possessing 8 and 37 unique spacer sequences ranging in length from 34 to 41 bp (Fig. 3). Although the arrays are separated by 2.1 Mbp within the *C. pasteurianum* genome, the 30 bp direct repeat sequences between the two CRISPR loci are identical, suggesting that the same set of Cas proteins are employed for spacer acquisition and interference. The 37-spacer locus was found to be associated with several *cas* genes (CP6013_0534–0541), while no such genes could be identified in proximity to the 8-spacer array. Based on the proposed classification of CRISPR-Cas systems^54^, the *C. pasteurianum* CRISPR system belongs to the Type I-B subtype owing to the presence of the signature Type I *cas3* gene (CP6013_0538) and Type I-B *cas8b (csh1*) gene (CP6013_0535). Furthermore, the *C. pasteurianum* CRISPR-Cas system possesses the same *cas* gene arrangement (*cas6*-*cas8b*-*cas7*-*cas5*-*cas3*-*cas4*-*cas1*-*cas2*) found in other prokaryotic Type I-B systems^54^. All eight *cas* genes are transcribed in the same direction and are located downstream of the 37-spacer CRISPR array. Analysis of similar Type I-B systems suggests that transcription occurs in the same direction as the *cas* genes, indicating existence of a CRISPR leader sequence possessing an active transcriptional promoter immediately upstream of the 37-spacer CRISPR array. We were unable to identify sequences at the 5′ or 3′ ends of the 8-spacer CRISPR array possessing homology to the presumed 37-spacer CRISPR leader. Accordingly, it is unclear if the 8-spacer CRISPR locus possesses a leader sequence, and, therefore, functionality of this CRISPR array is uncertain.

Processing of CRISPR RNAs (crRNAs) and subsequent binding to specific Cas proteins differs significantly between Type I and Type II CRISPR systems^54^. Type I and II crRNAs are first transcribed into a single large precursor RNA transcript (pre-crRNA), which is then cleaved into individual mature CRISPR RNAs by the ubiquitous RNase III enzyme in Type II systems^56^ and Cas6 in Type I systems^57^. crRNA processing involves a trans-activating RNA (tracrRNA) in Type II systems^56^, whereas Cas6 recognizes distinct RNA hairpin structures formed by the direct repeat sequence of Type I systems^57^. Mature Type I crRNAs possess a unique spacer sequence flanked by an 8 nt 5′ tag and a variable 3′ tag, both of which are derived from the CRISPR repeat sequence following processing^57^,^58^. The 5′ and 3′ tags of the crRNA are responsible for recognition by specific Cas proteins, while the unique internal spacer base-pairs to the target invading DNA and triggers endonuclease attack. Compared to Type I CRISPR-Cas machinery, interference against invading genetic elements is markedly simpler in Type II systems, which require only the Cas9 protein for endonucleolytic attack^54^,^59^. Type I systems are characterized by Cas3-mediated cleavage of invading targets^60^, which involves a multiprotein complex called Cascade (Cas complex for antiviral defence), comprised of Cas5, Cas6, Cas7, and Cas8^61^. The precise crRNA processing mechanism utilized by the Type I-B CRISPR systems from *C. thermocellum* and *Methanococcus maripaludis* have been recently elucidated^62^. Whereas 3′ ends of mature crRNAs were short and variable, indicating a lack of specificity in 3′ trimming by Cas6, 5′ ends possessed the trademark 8 nt tag of Type I crRNAs. Using Mfold^63^, we analyzed the direct repeat sequence from the Type I-B CRISPR system of *C. pasteurianum* and, as expected, identified a putative hairpin secondary structure (Fig. 3). Moreover, the 3′ end of the *C. pasteurianum* direct repeat sequence possesses 6/8 nucleotides in common with the 3′ terminus of the *C. thermocellum* repeat sequence corresponding to the 5′ tag of mature crRNA, suggesting a similar mechanism of processing between the two Type I-B systems^62^. In fact, the universal 8-nt 5′ tag, and CRISPR repeats in general, is often highly conserved between related bacteria^64^. Analysis of the *C. pasteurianum* direct repeat sequence by CRISPRFinder^55^ revealed CRISPR systems from a range of organisms with repeats possessing less than five mismatches to the *C. pasteurianum* query. *C. tetani* was the only organism identified that employs a repeat sequence identical to that of *C. pasteurianum*, suggesting that horizontal gene transfer potentially occurred between *C. tetani* and *C. pasteurianum*. However, no homology could be identified between spacers from the respective species. Other organisms with similar direct repeats include a range of clostridia (e.g., *C. botulinum*, *C. kluyveri*, and *C. autoethanogenum*), as well as *Bacillus coagulans* and *Eubacterium limosum*. Most CRISPR arrays harbored by these organisms specify only a small number of spacers (<7), compared to 37 in *C. pasteurianum*. It is likely that *C. pasteurianum* has been subjected to a greater degree of phage predation compared to other organisms employing similar CRISPR systems, leading to extensive acquisition of novel spacer sequences by *C. pasteurianum*. Although BLAST analysis of the 45 *C. pasteurianum* CRISPR spacers provided no perfect matches to potential protospacer sequences, a number of spacers returned protospacer hits with five or fewer mismatches, which could be sufficient to confer immunity, since spacer-protospacer sequences are often imperfect^65^. We are currently assessing the activity and functionality of the *C. pasteurianum* Type I-B CRISPR-Cas machinery against plasmid-borne protospacer sequences.

### Overview of central fermentative metabolism

**Figure 4:**
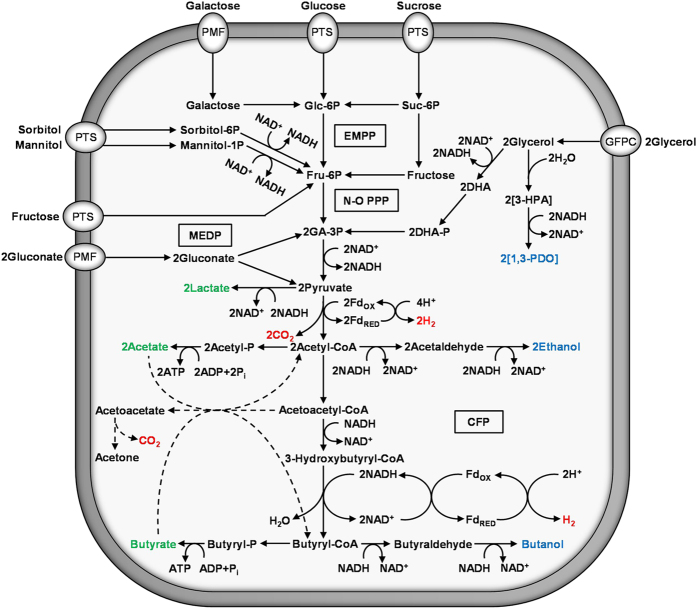
Overview of the central metabolic pathways of *C. pasteurianum* based on genomic analysis. Prevalent metabolic pathways leading to production of acids (green), alcohols (blue), and gases (red) are shown derived from commonly employed growth substrates. Many arrows represent multiple enzymatic conversions. The acetone formation pathway is depicted using dashed lines since acetone is not a common product of *C. pasteurianum* fermentations. The incomplete citrate cycle and other intermediary metabolic pathways are not depicted. Electron bifurcation by the Bcd-EtfAB enzyme complex is shown using 2NADH as reductant. Electron transfer via the EtfAB complex is not shown. Refer to main text for further discussion on central metabolic pathway enzymes and reactions. Abbreviations: EMPP, Embden-Meyerhof-Parnas pathway; N-O PPP, non-oxidative pentose phosphate pathway; MEDP, modified Entner-Doudoroff pathway; CFP, central fermentative pathways; PTS, phosphotransferase system; PMF, proton motive force; GFPC, glycerol facilitator protein channel; Glc, glucose; Suc, sucrose; Fru, fructose; DHA, dihydroxyacetone; 3-HPA, 3-hydroxypropionaldehyde; 1,3-PDO, 1,3-propanediol; GA, glyceraldehyde; FD_OX_, oxidized ferredoxin; FD_RED_, reduced ferredoxin.

The central fermentative metabolism of *C. pasteurianum* is unprecedented in nature, as the organism combines metabolic pathways found independently in other clostridia (Fig. 4). *C. pasteurianum* possesses the clostridial butyrate and butanol formation pathways, a characteristic of AB producers such as *C. acetobutylicum* and *C. beijerinckii*^16^,^24^,^66^. In contrast, the organism does not typically produce acetone, the chief co-product of the historic AB fermentative process. Furthermore, under certain culture conditions *C. pasteurianum* expresses a highly active 1,3-propanediol pathway in a manner similar to *C. butyricum*, which lacks an active butanol formation pathway^7^. Owing to this unique metabolic diversity, the central metabolism of *C. pasteurianum* is complex and highly substrate-dependent.

#### Primary upstream pathways

*C. pasteurianum* oxidizes sugars to pyruvate through the ubiquitous Embden-Meyerhof-Parnas (EMP) pathway (Fig. 4). The organization of EMP pathway genes in *C. pasteurianum* is similar to that found in *C. ljungdahlii*^67^ and other clostridia, where two gene clusters (CP6013_0364–0368: glyceraldehyde-3-phosphate dehydrogenase, phosphoglycerate kinase, triosephosphate isomerase, phosphoglycerate mutase, and enolase; and CP6013_0418 and CP6013_0419: 6-phosphofructokinase and pyruvate kinase) comprise the bulk of the EMP pathway genes. Unlike *C. ljungdahlii*, however, *C. pasteurianum* lacks the oxidative phase of the pentose phosphate pathway (PPP). A complete complement of genes corresponding to the non-oxidative phase of the PPP, including multiple copies of ribulose-5-phosphate isomerase (CP6013_0382, CP6013_2877, CP6013_3967), transketolase (CP6013_0396, CP6013_2326, CP6013_2327), and transaldolase (CP6013_2291, CP6013_2325, CP6013_2340), were identified in the genome. Like most Gram-positive bacteria, *C. pasteurianum* also lacks a full Entner-Doudoroff (ED) pathway. Instead, gluconate is catabolized via a modified ED pathway through conversion into 2-keto-3-deoxy-6-phosphogluconate, which is subsequently siphoned into glycolysis via glyceraldehyde-3-phosphate and pyruvate (see section below on gluconate fermentation)^68^. In line with other clostridia (e.g., *C. acetobutylicum*, *C. ljungdahlii*, *C. autoethanogenum*)^25^,^67^,^69^, *C. pasteurianum* possesses a non-cyclic, or branched, citrate “cycle”. Cell-free extracts have been shown to generate glutamate from oxaloacetate^70^, representing one branch of the pathway, which is comprised of citrate synthase, aconitase (CP6013_2146), and isocitrate dehydrogenase (CP6013_1709). Although we were unable to locate a gene corresponding to citrate synthase within the genome of *C. pasteurianum*, it is assumed that glutamate formation from oxaloacetate proceeds via the aforementioned pathway. The second branch of the citrate cycle is exemplified by malate dehydrogenase (CP6013_0066 and CP6013_0670) and fumarate hydratase (CP6013_3554 and CP6013_3555). However, genes corresponding to α-ketoglutarate dehydrogenase, succinate dehydrogenase, and succinyl-CoA synthetase could not be identified in the genome of *C. pasteurianum*.

#### Glycerol catabolism and 1,3-propanediol-formation pathway

**Figure 5:**
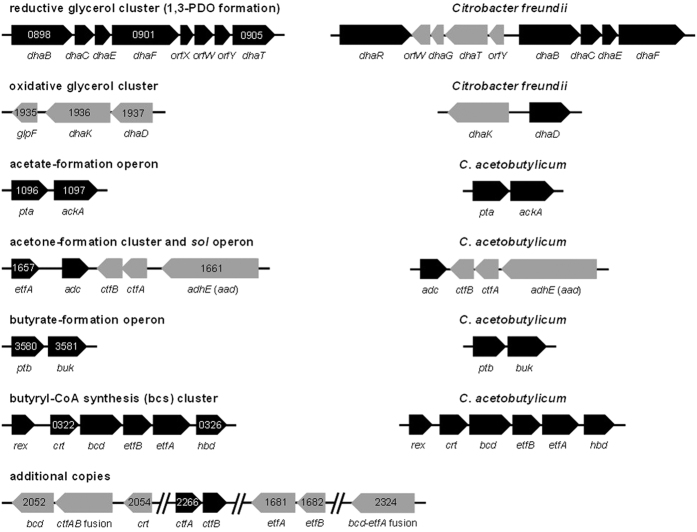
Genomic arrangement of key genes and operons involved in the central fermentative metabolism of *C. pasteurianum*. *C. pasteurianum* genes and operons (left) are compared with corresponding regulons from related species or key bacteria possessing similar metabolic pathways (right). Select additional copies of *C. pasteurianum* genes and operons are also depicted (bottom). Locus tags are provided for *C. pasteurianum* genes (CP6013 prefix is omitted). Metabolic functions of gene products are discussed in detail in the main text. Genes in black and grey depict different directions of transcription. All genes and intergenic regions are depicted to scale.

Compared to other fermentations carried out by *C. pasteurianum*, the fermentation of glycerol is unique owing to production of 1,3-propanediol, a signature product of glycerol metabolism^71^. Glycerol is catabolized by one of two divergent pathways, deemed the reductive and oxidative routes. The former pathway involves direct reduction of glycerol to 1,3-propanediol. Glycerol dehydratase (*dhaBCE*; CP6013_0898–0900) converts glycerol into the toxic intermediate, 3-hydroxypropionaldehyde, which is then reduced to 1,3-propanediol by 1,3-propanediol dehydrogenase (*dhaT*; CP6013_0905)^72^. In the *C. pasteurianum* genome, both enzymes are encoded within a 7 kb reductive glycerol regulon, the structure and organization of which is distinct from that of other organisms capable of growth on glycerol, such as *Citrobacter freundii*^73^ (Fig. 5). Both *dhaBCE* and *dhaT* genes from *C. pasteurianum* have been cloned and characterized^72^,^74^ and the corresponding proteins are highly conserved between *C. pasteurianum* and various species of *Klebsiella* and *Citrobacter* (65–81% identity). Further, DhaT from *C. pasteurianum* was found to share 86% of amino acid identities with the same enzyme from *C. butyricum*. The reductive 1,3-propanediol pathway requires NADH, in addition to vitamin B_12_^75^, and offers a non-glycolytic route for consuming excess reducing equivalents^76^. Glycerol is more reduced than biomass, and thus, leads to a net production of NADH when biomass is formed from glycerol under anaerobic conditions^1^. Whereas flux through glycolytic pathways, such as the butanol and ethanol formation routes, results in redox balance, the NADH-consuming 1,3-propanediol pathway is the only metabolic route that affords the cell a means of oxidizing the surplus of reducing equivalents derived from biomass formation. In fact, the ability to ferment glycerol as a sole source of energy is a metabolic feature exclusive to anaerobic organisms possessing an active 1,3- or 1,2-propanediol-producing pathway^77^. The alternative oxidative route of glycerol catabolism in *C. pasteurianum* involves conversion of glycerol into the glycolytic intermediate dihydroxyacetone phosphate by the concerted action of glycerol dehydrogenase (DhaD) and dihydroxyacetone kinase (DhaK). Dihydroxyacetone phosphate is then further oxidized to pyruvate via the standard glycolytic pathway. The genome of *C. pasteurianum* encodes at least five putative *dhaD* genes (CP6013_0378, CP6013_1584, CP6013_1937, CP6013_3371, and CP6013_3819) and one potential *dhaK* gene (CP6013_1936), whereby the chief *dhaDK* regulon (CP6013_1936 and CP6013_1937) precedes a *glpF* gene (CP6013_1935) encoding the glycerol uptake facilitator protein (Fig. 5). An abundance of glycerol-catabolizing enzymes presumably enables *C. pasteurianum* to tolerate exceptionally high concentrations of the substrate (up to 170 g L^−1^) without detectable growth inhibition^5^.

#### Lactate- and hydrogen-formation pathways

Although the preferred outcome of pyruvate catabolism in *C. pasteurianum* involves oxidation to acetyl-CoA, certain culture conditions can result in significant accumulation of lactate via direct reduction of pyruvate by NADH, catalyzed by lactate dehydrogenase^1^,^2^,^5^. Alcohol dehydrogenases involved in production of ethanol and butanol contain iron, and therefore, conditions of iron limitation have been shown to impede alcohol production and trigger lactate formation^5^. In this sense, the lactate pathway operates as a backup valve to relieve the cell of excess reductant when preferred routes of NADH oxidation, such as ethanol and butanol production, are blocked. Like *C. cellulolyticum*^78^, the genome of *C. pasteurianum* harbors two l-lactate dehydrogenase genes (CP6013_1427 and CP6013_0421), which share 43% of amino acid identities. Under standard growth conditions where iron is not limiting, pyruvate is oxidized to acetyl-CoA through pyruvate:ferredoxin oxidoreductase, referred to as the phosphoroclastic reaction owing to the formation of ATP and acetate from acetyl-CoA during growth on glucose^79^. The genome of *C. pasteurianum* contains three putative pyruvate:ferredoxin oxidoreductase genes (CP6013_1431, CP6013_2634, CP6013_1432). The organism also harbors a potential alternative route of pyruvate oxidation via pyruvate formate lyase^80^, for which three genes are present in the genome (CP6013_3048, CP6013_2343, CP6013_2339). It has been suggested, however, that the pyruvate formate lyase reaction is predominantly anabolic in *Clostridium*^81^, rendering pyruvate:ferredoxin oxidoreductase the prevalent pathway of pyruvate oxidation. Electrons generated from the phosphoroclastic system are utilized to reduce ferredoxin, which is primarily oxidized by hydrogenase with coupled evolution of molecular hydrogen^5^. Ferredoxin (CP6013_3660) was first discovered in *C. pasteurianum*^82^ and has since served as a model electron transfer protein. A total of three ferredoxin iron hydrogenase-encoding genes (CP6013_3094, CP6013_3784, CP6013_3422) can be identified in the genome of *C. pasteurianum*, including the bidirectional hydrogenase I (CP6013_3094)^83^ and the H_2_-oxidizing, uptake hydrogenase (CP6013_3784 or CP6013_3422)^84^.

#### Acetate-formation pathway

Acetyl-CoA embodies the central branch point of clostridial fermentations for direct conversion into acetate and ethanol, or condensation to yield butyrate and butanol^16^,^66^. As discussed in detail below (refer to section on substrate redox considerations), the fate of acetyl-CoA in *C. pasteurianum* is largely substrate-dependent and dictated by redox. The cell relies on the analogous acetate- and butyrate-formation pathways as the major source of ATP synthesis, since production of either metabolite results in substrate-level phosphorylation (Fig. 4). *C. pasteurianum* harbors a single acetate-formation operon comprised of *pta* (CP6013_1096) and *ackA* (CP6013_1097) encoding phosphoacetyltransferase and acetate kinase, respectively. Interestingly, the coding sequences corresponding to *pta* and *ackA* in *C. pasteurianum* are separated by 134 bp, whereas the analogous genes in *C. acetobutylicum* are separated by only 11 bp (Fig. 5). Since a putative promoter could be identified in the 134 bp intergenic region, these two genes may not exist in an operon structure, which contrasts the genetic arrangement found in most clostridia^85^. This finding may extend to other strains of *C. pasteurianum*, as the *pta* and *ackA* genes of strain BC1 also possess a relatively large spacer region of 103 bp.

#### Ethanol-formation pathway

When *C. pasteurianum* is grown on reduced substrates, the organism relies at least partially on the ethanol formation pathway to maintain redox balance. Ethanol production has been shown to correlate with pH between values of 6.5 and 7.5 during batch fermentations of glycerol^1^. Overall, however, ethanol production plays only a minor role in most fermentations carried out by *C. pasteurianum*, since the organism prefers the butyrate- and butanol-formation pathways for regeneration of NAD^+^. The genome of *C. pasteurianum* harbors an array of genes encoding aldehyde dehydrogenases (CP6013_0292, CP6013_1611, CP6013_1661, and CP6013_2575) and alcohol dehydrogenases (CP6013_0781, CP6013_1579, CP6013_2048, CP6013_2062, CP6013_3785, and CP6013_2711) for reduction of acetyl-CoA to ethanol via acetaldehyde. *C. acetobutylicum* harbors two bifunctional aldehyde-alcohol dehydrogenases, encoded by *adhE (aad*) and *adhE2*, that play major roles in the production of butanol and, to a lesser extent, ethanol^86^,^87^,^88^. Four protein products encoded in the genome of *C. pasteurianum* (CP6013_0292, CP6013_1611, CP6013_1661, and CP6013_2575) were found to possess substantial similarity (62–81%) to both AdhE and AdhE2 from *C. acetobutylicum*. It is probable that genes encoding these enzymes are involved in the production of ethanol and butanol in *C. pasteurianum*.

#### C_4_ trunk pathway

Butyrate- and butanol-forming clostridia produce C_4_ metabolites through the condensation of two molecules of acetyl-CoA^16^. This complex transformation involves the sequential action of four enzymes: thiolase (acetyl-CoA acetyltransferase; *thl*), 3-hydroxybutyryl-CoA dehydrogenase (*hbd*), crotonase (3-hydroxybutyryl-CoA dehydratase; *crt*), and butyryl-CoA dehydrogenase (*bcd*), and results in the generation of butyryl-CoA and oxidation of two moles of NADH per mole of butyryl-CoA formed. Thiolase, the first enzyme of this trunk pathway, condenses two molecules of acetyl-CoA, yielding acetoacetyl-CoA. *C. pasteurianum* harbors two putative thiolase genes (CP6013_2289 and CP6013_3617), one of which (CP6013_3617) has been cloned and characterized^89^. The two thiolase protein sequences share 90% of amino acid identities. Genes involved in the conversion of acetoacetyl-CoA to butyryl-CoA are organized in an operon in *C. acetobutylicum*, referred to as the butyryl-CoA synthesis (bcs) operon (Fig. 5). In addition to *crt*, *bcd*, and *hbd*, the operon also encodes both electron-transfer flavoprotein (Etf) subunits, *etfA* and *etfB*, required for reduction of crotonyl-CoA by NADH in the enzymatic step catalyzed by Bcd^90^. The full five-gene operon (*crt*-*bcd*-*etfB*-*etfA*-*hbd*; CP6013_0322–0326) was found to be highly conserved between *C. pasteurianum* and *C. acetobutylicum* (80% nucleotide identity across the entire 4.8 kb operon), as well as most other clostridia. Based on amino acid identities, the proteins of the bcs operon are most similar to those from *C. arbusti*, *C. acetobutylicum*, *C. tetani*, and *C. botulinum* (69–94% amino acid identity). In particular, 92–94% of amino acids of the bcs enzymes are common between *C. pasteurianum* and *C. arbustii*. In addition to the bcs operon, additional copies of *crt* (CP6013_2054), *bcd* (CP6013_2052 and CP6013_2324), *etfB* (CP6013_1682), *etfA* (CP6013_1681, CP6013_1657, and CP6013_2324), and *hbd* (CP6013_1378 and CP6013_1968), could be identified in the genome of *C. pasteurianum* (Fig. 5). Multiple copies of the bcs operon genes have been reported in other solventogenic clostridia, including *C. carboxidivorans* and *C. beijerinckii*^91^. In addition to the bcs operon, we also identified the *rex* gene (CP6013_0321) encoding a putative redox-sensing transcriptional regulator upstream of the bcs operon in *C. pasteurianum*. The *C. pasteurianum* Rex protein was found to possess 76% identity to the corresponding protein from *C. acetobutylicum*^92^. Accordingly, it appears that Rex-associated regulation of the bcs operon is similar between these organisms and is dictated by the cellular NADH/NAD^+^ ratio.

Growing cultures of *C. pasteurianum* generate reductant in the form of NADH and reduced ferredoxin^93^. Theoretically, electrons can be shuttled between these two species via ferredoxin:NAD^+^ oxidoreductase/NADH:ferredoxin oxidoreductase, which catalyzes the reversible reduction of NAD^+^ by reduced ferredoxin^93^,^94^,^95^. Electron flow from ferredoxin to NAD^+^ is evident under certain non-standard culture conditions, such as inhibition of hydrogenase by carbon monoxide^5^ or methyl viologen^96^. In these instances, abundant NADH is utilized to drive production of reduced end products, typically butyrate and butanol^5^. However, the ferredoxin:NAD^+^ oxidoreductase reaction is inhibited by low levels of NADH^93^,^95^, rendering the NADH:ferredoxin oxidoreductase pathway the presumed direction of electron flux in clostridial fermentations. Still, electron flow from NADH (E_0_′ = −320 mV) to ferredoxin (E_0_′ = −400 mV), is highly unfavorable, spawning considerable skepticism surrounding the thermodynamic feasibility of this pathway *in vivo*^93^. Despite this uncertainty, it has been observed that glucose-grown cultures of *C. pasteurianum* evolve more molecular hydrogen than can be accounted for by the phosphoroclastic reaction (determined by the combined amount of acetate and butyrate formed), indicating that under certain conditions NADH serves as reductant through operation of the unfavorable NADH:ferredoxin oxidoreductase reaction^7^. Likewise, it has been shown that cell-free extracts of *C. pasteurianum* produce hydrogen gas from acetyl-CoA and NADH, again implying electron transfer from NADH to ferredoxin^94^. This thermodynamic mystery has remained unresolved for more than 35 years, until recently when Hermann *et al*.^97^ proposed that ferredoxin reduction by NADH proceeds via coupling to the exergonic reduction of crotonyl-CoA to butyryl-CoA by NADH. This theory opened the door to a novel mode of energy conservation through electron bifurcation by the Bcd-EtfAB enzyme complex in *C. pasteurianum*, *C. kluyveri*, and possibly other solventogenic clostridia^98^. EtfAB has been implicated as the key enzyme complex responsible for electron bifurcation^97^, whereby one electron of NADH is utilized for the exergonic reduction of crotonyl-CoA to butyryl-CoA and the free enthalpy change is harnessed to drive reduction of ferredoxin using the remaining electron from NADH. Repeating this process consumes two moles of NADH and generates one mole each of butyryl-CoA and reduced ferredoxin^97^. The resulting electrons from ferredoxin are then used to drive production of molecular hydrogen by the hydrogenase enzyme, at last providing an explanation for earlier biochemical data obtained using cell-free extracts of *C. pasteurianum*^94^. Interestingly, gene CP6013_2324 within the genome of *C. pasteurianum* was found to possess similarity to both *bcd* (CP6013_0323) and *etfA* (CP6013_0325), suggesting presence of a Bcd-EtfA fusion protein. It has been suggested that redox partners evolve into a single fusion protein to promote more efficient conversion of unstable intermediates and rapid transfer of electrons^99^. A similar *bcd*-*etfA* fusion ortholog could only be identified in *C. kluyveri* (76% nucleotide identity), which could provide insight into the recently-proposed electron bifurcation mechanism of the Bcd-EtfAB enzyme complex in *C. pasteurianum* and *C. kluyveri*^97^,^98^.

#### Acetone-formation pathway

*C. pasteurianum* harbors a full acetone-formation pathway consisting of CoA transferase subunits A and B (encoded by *ctfAB*) and an acetoacetate decarboxylase (encoded by *adc*) for conversion of acetoacetyl-CoA to acetone via acetoacetate^37^,^42^ (Fig. 5). The structure and arrangement of this classical acetone-forming sol operon are identical to that of *C. acetobutylicum*^100^, whereby the *ctfAB* genes (CP6013_1660 and CP6013_1659, respectively) are preceded by a putative *adhE (aad*) gene (CP6013_1661). Additional copies of the *ctfAB* genes (CP6013_2266 and CP6013_2267, respectively) and two genes encoding putative CtfAB fusion proteins (CP6013_2053 and CP6013_3216) were also identified in the genome (Fig. 5). Analysis of genes CP6013_2053 and CP6013_3216 identified similar *ctfAB* fusion genes in a number of clostridia, including *C. beijerinckii*, *C. carboxidivorans*, and *C. saccharobutylicum*. In *C. pasteurianum*, the sol operon is positioned adjacent to a reverse-orientation *adc* gene (CP6013_1658), as found in *C. acetobutylicum*^100^ (Fig. 5). The *C. pasteurianum* CtfAB and Adc enzymes possess a high degree of similarity (71–84%) to the corresponding proteins of *C. acetobutylicum*, a significant acetone-producer. Despite these similarities, acetone is not a common metabolite of *C. pasteurianum*^101^. Production of acetone, as well as ethanol and butanol, is inherently linked to acetate and butyrate uptake in *C. acetobutylicum* as a means of preventing acid crash under low pH conditions^102^. Consequently, a lack of acetone production could be the result of an inability of *C. pasteurianum* to uptake and reassimilate acids, as acid levels generally increase throughout the course of fermentation^1^,^103^ and do not exhibit the characteristic drop associated with acetate and butyrate assimilation by *C. acetobutylicum*. It is also possible that the acetone pathway remains inactive in *C. pasteurianum* due to a lack of pathway induction under standard growth conditions, poor enzymatic activities, or lack of a functional transcriptional promoter to drive expression of the *sol* operon or *adc* gene. Induction of acetone production in *C. acetobutylicum* has been studied extensively and inducers include low pH and elevated concentrations of acetate and butyrate^104^.

#### Butyrate- and butanol-formation pathways

Similar to acetyl-CoA, butyryl-CoA serves as a major branch point in the central metabolism of *C. pasteurianum*. Butyryl-CoA can be converted into butyrate or further reduced to butanol in pathways that mimic the C_2_ fermentative pathways leading to production of acetate and ethanol. *C. pasteurianum* harbors a single butyrate-formation operon, consisting of phosphotransbutyrylase (*ptb*; CP6013_3580) upstream of butyrate kinase (*buk*; CP6013_3581) (Fig. 5). Ptb and Buk from *C. pasteurianum* possess a high degree of similarity (80% and 73%, respectively) to the corresponding enzymes from *C. acetobutylicum*. Unlike *C. acetobutylicum*, however, we were unable to identify a second copy of *buk* [i.e. *buk2*^105^] within the genome of *C. pasteurianum*. The other pathway from butyryl-CoA is the reductive butanol formation route, where consecutive dehydrogenation steps convert butyryl-CoA first to butyraldehyde, then butanol. In addition to the aforementioned *adhE (aad*) and *adhE2* genes, two butanol dehydrogenases, encoded by *bdhA* and *bdhB*, have been implicated in butanol formation in *C. acetobutylicum*^106^. BLAST analysis of the *C. pasteurianum* genome using BdhA and BdhB protein queries returned a large array of alcohol dehydrogenases (CP6013_2711, CP6013_1579, CP6013_2048, CP6013_0905, CP6013_2062, CP6013_1661, CP6013_3785, CP6013_0292, CP6013_0781, CP6013_2575, and CP6013_1611) possessing similarity to the *C. acetobutylicum* isozymes. Notably, protein products corresponding to genes CP6013_2711 and CP6013_1579 produced the highest degree of identity to both BdhA (72% and 41%, respectively) and BdhB (67% and 39%, respectively) from *C. acetobutylicum*. Although *bdhA* and *bdhB* occur in tandem within the chromosome of *C. acetobutylicum*^107^, alcohol dehydrogenases possessing a similar genetic arrangement could not be identified in the genome of *C. pasteurianum*. Surprisingly, disruption of *bdhA* or *bdhB* in *C. acetobutylicum* had no effect on solvent formation, while disruption of *adhE* nearly abolished production of solvents^86^. Based on these findings, genes possessing the greatest protein identity to *adhE* from *C. acetobutylicum*, specifically CP6013_1661, CP6013_2575, CP6013_0292, and CP6013_1611, are likely to be the greatest contributors to butanol, as well as ethanol, formation in *C. pasteurianum*.

### Effect of substrate reductance on fermentation end product distribution

**Figure 6:**
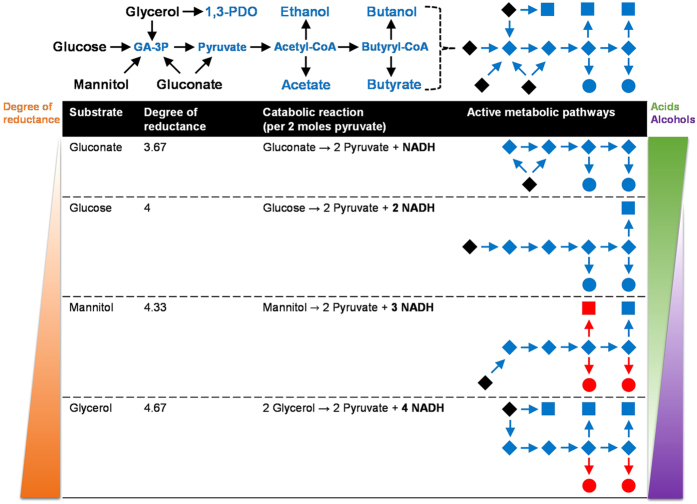
Effect of substrate degree of reductance on the fermentation product profile of *C. pasteurianum*. Active metabolic pathways employed by the cell are shown during growth on a range of substrates possessing varied degrees of reductance. General catabolic equations are provided and show the number of moles of reducing equivalents generated (in bold) per two moles of pyruvate formed. Substrates and pathway intermediates are depicted as black and blue diamonds, respectively, while acid and alcohol products are shown as blue circles and squares, respectively. Trace products (<1 g L^−1^) are shown in red. Product titers are provided and discussed within the main text. Lactate and acetone were not detected and gaseous products were not measured. Abbreviations are defined in Fig. 4.

*C. pasteurianum* readily utilizes glucose, fructose, mannitol, sorbitol, and sucrose, among other substrates^108^ (Fig. 4). Substrates that are metabolized at a reduced rate include arabinose, galactose, lactose, starch, and xylose. *C. pasteurianum* genes encoding putative phosphotransferase system (PTS) components could be identified for most of these fermentable substrates^109^,^110^, while galactose and gluconate have been shown to be taken up using a proton motive force (PMF)^111^. Owing to the immense substrate range exhibited by *C. pasteurianum*, product distribution varies dramatically and is dictated foremost by the degree of reductance of the substrate^112^. Such an effect has been documented for *C. pasteurianum*, where fermentation of glucose generates a predominantly acidogenic metabolism, while fermentation of mannitol or glycerol yields almost exclusively alcohols^5^,^7^. Based on our genome sequencing data, we further probed this model by comparing product distribution of *C. pasteurianum* grown on substrates of varying degrees of reductance, thereby allowing manipulation of the intracellular NADH/NAD^+^ ratio^113^. We selected gluconate, glucose, mannitol, and glycerol and show below that catabolism of these substrates leads to four distinct fermentation profiles ranging from entirely acidogenic to primarily alcohologenic (Fig. 6). In each case, cell growth and product distribution were assessed by analyzing 60–90 h fermentation samples from anaerobic static flask cultures at pH 6.0–6.2 containing 40 g L^−1^ of substrate (sodium gluconate, glucose, mannitol, or glycerol).

With a degree of reductance of 3.67, gluconate is the most oxidized substrate fermented by *C. pasteurianum*. The substrate is first dehydrated to 2-keto-3-deoxy-gluconate (KDG) by gluconate dehydratase (CP6013_2550)^114^, followed by phosphorylation to 2-keto-3-deoxy-6-phosphogluconate by 2-keto-3-deoxygluconokinase (CP6013_3201). The resulting product is then cleaved by 2-keto-3-deoxy-6-phosphogluconate aldolase (CP6013_3200 and CP6013_2554)^68^, yielding glyceraldehyde-3-phosphate and pyruvate (Fig. 4). Catabolism of one mole of gluconate generates two moles of pyruvate, yet only one mole of NADH (Fig. 6). *C. pasteurianum* static flask cultures grown on sodium gluconate yielded an entirely acidogenic fermentative metabolism yielding 8.5 ± 0.6 g L^−1^ acetate and 5.9 ± 0.3 g L^−1^ butyrate, as the butyrate pathway alone was sufficient to oxidize NADH. The NADH-consuming ethanol and butanol pathways were not induced during gluconate catabolism, presumably due to a low intracellular NADH/NAD^+^ ratio. With a degree of reductance of 4^115^, glucose is less oxidized than gluconate, resulting in the production of two moles of NADH per two moles of pyruvate generated (Fig. 6). Fermentation of glucose often yields exclusively acetate and butyrate, yet some studies have reported notable butanol production^101^. We observed butanol (3.4 ± 1.2 g L^−1^) as the predominant fermentation product, with equal quantities of acetate (2.4 ± 0.4 g L^−1^) and butyrate (2.4 ± 0.8 g L^−1^). The relatively high levels of butanol detected in this study may be explained by growth medium formulation, as we utilized a medium optimized for production of butanol from glycerol^103^. Mannitol and sorbitol, both six-carbon sugar alcohols that are readily fermented by *C. pasteurianum*^112^, possess degrees of reductance of 4.33^115^, and therefore, are more reduced than glucose. Both substrates enter the cell using the same PEP-dependent PTS where they are phosphorylated and converted into fructose-6-phosphate (Fig. 4) by mannitol-1-phosphate dehydrogenase (CP6013_0304 and CP6013_2639) or sorbitol-6-phosphate dehydrogenase (CP6013_0284 and CP6013_0306)^109^,^110^,^116^. Oxidation of mannitol or sorbitol generates a total of three moles of NADH per two moles of pyruvate formed (Fig. 6). Fermentation of mannitol by *C. pasteurianum* leads to a product profile characterized by high butanol selectivity, as cultures produced 6.0 ± 1.7 g L^−1^ butanol and only trace amounts of acetate (0.4 ± 0.1 g L^−1^), butyrate (0.5 ± 0.3 g L^−1^), and ethanol (0.7 ± 0.4 g L^−1^), indicating that the cell relies almost exclusively on the butanol pathway for oxidation of NADH. Similar products have been detected from the fermentation of mannitol by *C. pasteurianum* in continuous culture^112^. Since glycerol possesses a degree of reductance of 4.67^115^, glycerol produces four moles of NADH per two moles of pyruvate formed (Fig. 4), compared to only three moles of NADH from mannitol or sorbitol (Fig. 6). Glycerol is taken up by *C. pasteurianum* using a unique glycerol facilitator protein channel (CP6013_1935), where it is then converted into 1,3-propanediol or siphoned into glycolysis via dihydroxyacetone phosphate. Owing to its high degree of reduction, glycerol catabolism by *C. pasteurianum* leads to substantial quantities of reduced end products, specifically butanol and 1,3-propanediol^1^,^5^. Medium formulation and cultivation conditions can be manipulated to favor production of either product^103^,^117^. Under the conditions employed in this study, butanol titer (7.0 ± 0.2 g L^−1^) surpassed that of 1,3-propanediol (5.2 ± 1.8 g L^−1^), while ethanol (1.3 ± 0.4 g L^−1^), acetate (0.7 ± 0.2 g L^−1^), and butyrate (0.2 ± 0.1 g L^−1^) represented minor co-products. The highly reduced product profile of *C. pasteurianum* during growth on glycerol underscores the immense industrial potential of this organism in producing butanol from crude glycerol. Since substantial quantities of 1,3-propanediol are produced along with butanol, the 1,3-propanediol pathway represents a key target of rational metabolic engineering. Note that fundamental genetic engineering technologies have only recently been developed for this organism^31^,^32^,^33^, whereas previous efforts have focused on random mutagenesis^28^,^118^ and bioprocessing approaches^28^, such as separation of butanol and 1,3-propanediol product streams via *in situ* removal of butanol.

While the redox state of the fermentation substrate represents the chief factor governing product distribution in *C. pasteurianum*, carbon and electron flow can be manipulated using a number of strategies. The effect of carbon monoxide on anaerobic fermentations has been widely documented in *Clostridium*^119^, where controlled gassing leads to potent inhibition of the hydrogenase enzyme. With hydrogen production shut down, cells are forced to utilize the ferredoxin:NAD^+^ oxidoreductase reaction to oxidize ferredoxin, resulting in NADH formation and subsequent production of butanol and ethanol^5^. In addition to carbon monoxide, redox dyes, such as methyl viologen, can be employed to induce solvent production in the clostridia, also through inhibition of the hydrogenase enzyme^96^. Finally, it has recently been shown that *C. pasteurianum* is able to utilize electrons derived directly from a supplied electric current^4^. Whereas other electroactive organisms require an exogenous mediator to facilitate electron transfer, *C. pasteurianum* is a rare exception capable of uptaking electrons directly from a cathode. Moreover, cells were found to utilize substrate and exogenous electrons concomitantly, thus building on the biotechnological potential harnessed by *C. pasteurianum*. On the other hand, utilization of externally-supplied electrons manifested in increased titers of 1,3-propanediol, rather than butanol, which is in line with the role of the 1,3-propanediol pathway in maintaining redox poise^76^. As butanol is the most promising end product of *C. pasteurianum* metabolism, electrosynthesis of butanol represents an important and challenging target of future strain engineering efforts. In this context, it is anticipated that the genomic analysis presented herein, as well as previous studies of *C. pasteurianum* metabolism^1^,^5^ and still-developing genetic technologies^34^, will lead to productive metabolic engineering outcomes and robust mutant strains for industrial conversion of waste glycerol to butanol using *C. pasteurianum*.

## Materials and Methods

### Strain, oligonucleotides, and growth conditions

*C. pasteurianum* type strain ATCC 6013 (DSM 525) was obtained from the American Type Culture Collection (ATCC). Oligonucleotides (see Supplementary Table S3) were purchased from and synthesized by Integrated DNA Technologies (IDT; Coralville, IA) at the 25 nanomole scale using standard desalting. All chemicals were purchased from Sigma-Aldrich (St. Louis, MO). Strain ATCC 6013 was grown under strictly anaerobic conditions in a semi-defined medium^1^,^103^ containing per liter: 22 g KH_2_PO_4_, 6.68 g K_2_HPO_4_, 7.35 g (NH_4_)_2_SO_4_, 5.08 g Bacto yeast extract, 0.2 g MgSO_4_ · 7H_2_O, 0.02 g CaCl_2_ · 2H_2_O, 0.06 g FeSO_4_ · 7H_2_O, 1 mg resazurin, and 2 ml trace element solution SL 7. The initial pH of the medium was 6.0–6.1 prior to sterilization. Carbon sources (sodium gluconate, glucose, mannitol, and glycerol) were sterilized separately as 100 g L^−1^ stock solutions and added to culture flasks to achieve a final concentration of 40 g L^−1^. Cysteine-HCl (0.5 g L^−1^) was used to reduce growth medium prior to inoculation. Static cultures were grown in 125 ml Erlenmeyer flasks containing 50 ml medium within an anaerobic containment chamber (Plas-Labs, Inc.; Lansing, MI) consisting of an environment of 85% N_2_, 10% H_2_, and 5% CO_2_. Seed cultures were prepared by heat-shocking single sporulated agar plate colonies at 80 °C for 10 minutes in 10 ml 2×YTG medium, pH 6.4 (16 g L^−1^ Bacto tryptone, 10 g L^−1^ Bacto yeast extract, 5 g L^−1^ glucose, and 4 g L^−1^ NaCl) as described previously^31^,^32^.

### Analytical methods

Cell growth was monitored by measuring optical density at 600 nm (OD_600_). Culture supernatants were analyzed for metabolite production 60–90 h after inoculation. Product concentrations were determined by LC-10AT HPLC analysis (Shimadzu; Kyoto, Japan) equipped with a RID-10A refractive index detector (Shimadzu; Kyoto, Japan) and Aminex HPX-87H column (Bio-Rad Laboratories; Richmond, CA). Column temperature was maintained at 65 °C. The mobile phase consisted of 5 mM H_2_SO_4_ (pH 2.0) at a flow rate of 0.6 mL min^−1^. RID signal data processing was performed using Clarity Lite (DataApex; Prague, Czech Republic). End product titers reported represent the average of two or three biological replicates.

### Phage induction and transmission electron microscopy

Phage excision and transmission electron microscopy (TEM) were performed in a manner similar to previous methods^27^. For mitomycin C induction, a single sporulated colony of *C. pasteurianum* ATCC 6013 was heat-shocked, grown to exponential phase, and used to inoculate 200 ml of fresh 2×YTG medium, pH 6.4. The resulting culture was grown to early exponential phase (OD_600_ 0.2–0.3) and divided into six 25 ml cultures. Mitomycin C was added to a final concentration of 0, 0.5, 1, 2.5, 5, or 10 μg ml^−1^ and OD_600_ was monitored until a sharp decline in turbidity was observed approximately 4 h post-induction. One ml of the resulting phage lysates was centrifuged at 10,000× g for 10 minutes and the supernatants were filtered through a 0.45 μm filter. Following washing of phage particles twice with 0.1 M ammonium acetate, pH 7.5, five μl of lysate was pipetted onto 200-mesh Formvar/carbon-coated copper grids and incubated for approximately five minutes. Excess lysate was blotted with Whatman filter paper and grids were allowed to dry overnight. Grids were then stained for 10 minutes using a saturated uranyl acetate solution, followed by washing with 50% ethanol and drying in air for approximately three hours. Imaging was performed at 60 kV using a Philips CM10 transmission electron microscope equipped with a digital camera. Phage images were captured using 245,000× magnification.

### DNA isolation, sequencing, and analysis

Total DNA was isolated from *C. pasteurianum* ATCC 6013 according to a previous method^32^ using a Qiagen (Valencia, CA) DNeasy Blood and Tissue Kit. The genome of *C. pasteurianum* ATCC 6013 was sequenced, assembled, and annotated as described^37^. An additional two single molecule real-time (SMRT) cells were sequenced from a size-selected large insert library using the RS II analyzer (Pacific Biosciences; Menlo Park, CA). Methylome analysis was performed by Pacific Biosciences and the Genomic Resource Center at the Institute for Genome Sciences (University of Maryland School of Medicine; Baltimore, MD) using raw RS II sequencing reads and the *C. pasteurianum* draft genome^37^ as a reference.

Long range PCR (15–35 kb) was performed using LongAmp *Taq* DNA Polymerase (New England Biolabs; Ipswich, MA). Large PCR products and intact genomic DNA were separated using 0.3–0.5% agarose gels and low voltage (12–15 V) electrophoresis for 12–18 h. Restriction endonucleases were obtained from New England Biolabs (Ipswich, MA) and utilized according to the manufacturer’s guidelines.

## Additional Information

**Accession numbers:** This Whole Genome Shotgun project has been deposited at GenBank under the accession JPGY00000000. The version described in this paper is version JPGY02000000.

**How to cite this article**: Pyne, M. E. *et al*. Genome-directed analysis of prophage excision, host defence systems, and central fermentative metabolism in *Clostridium pasteurianum*. *Sci. Rep.* **6**, 26228; doi: 10.1038/srep26228 (2016).

## Supplementary Material

Supplementary Information

## Figures and Tables

**Table 1:** Overview of *C. pasteurianum* genome sequencing projects completed or currently underway.

**General features**	**Type strain (ATCC 6013 = DSM 525)**				**Non-type strains**	
	This study	ATCC 6013^36^	DSM 525^35^	DSM 525^42^	BC1 (unpublished data)	NRRL B-598^41^
Status	1 linear contig	Complete	Complete	37 contigs	Complete	138 contigs
Size (bp)	4,373,654	4,351,893	4,352,101	4,285,687	4,990,707	6,041,878
GC content (%)	29.9	29.9	30	29.8	30.6	29.6
Plasmid	ND	ND	ND	ND	pCLOPA01 (53,393 bp)	ND
Protein-coding genes	3,803	3,791	3,791	3,766	4,463	5,057
Function prediction	3,039	3,220	3,220	NR	NR	NR
Hypothetical	1,006	768	768	NR	NR	NR
tRNA genes	81	81	81	74	74	76
rRNA genes	30	30	30	3	27	29

ND: not detected. NR: not reported.

**Table 2:** Overview of the *C. pasteurianum* methylome and RM systems.

**Methylation motif (5**′**-3**′)^a^	**Methylation type**^b^	**Number of methylated sites detected (percentage)**	**Corresponding RM activity**	**Putative annotation**	**Reference**
GA**T**C	m6A	5,105/13,630 (37.4%)	CpaI (Type II)	R: CP6013_0098	18
				M: CP6013_0095/	
				CP6013_0096/	
				CP6013_0097	
AAGNNNNNC**T**CC	m6A	487/524 (92.9%)	CpaAII (Type I)	R: CP6013_0336	32
				M: CP6013_0337	
				S: CP6013_0338	
CAAAAAR	m6A	945/3,945 (24.0%)	NI	M: CP6013_1459	This study^21^
GRTAAAG	m6A	987/2,612 (37.8%)	NI	RM: CP6013_0727	This study^21^
Inactive	Inactive	Inactive	NI	M: CP6013_0738	21
CGCG	m5C	ND	CpaAI (Type II)	R: CP6013_2557	52
				M: CP6013_2558	

ND: not detected; NI: not identified.

^a^Methylated bases in the provided (forward) and reverse strand are underlined and bolded, respectively. Methylation of the CpaAI recognition sequence has not been determined.

^b^Only m6A base methylations were detected in this study.

## References

[b1] Biebl H. Fermentation of glycerol by *Clostridium pasteurianum* - Batch and continuous culture studies. J. Ind. Microbiol. Biotechnol. 27, 18–26, doi: 10.1038/sj.jim.7000155 (2001). 11598806

[b2] Taconi K. A., Venkataramanan K. P. & Johnson D. T. Growth and solvent production by *Clostridium pasteurianum* ATCC (R) 6013 (TM) utilizing biodiesel-derived crude glycerol as the sole carbon source. Environ. Prog. Sustain. Energy 28, 100–110, doi: 10.1002/ep.10350 (2009).

[b3] Jensen T. O., Kvist T., Mikkelsen M. J., Christensen P. V. & Westermann P. Fermentation of crude glycerol from biodiesel production by *Clostridium pasteurianum*. J. Ind. Microbiol. Biotechnol. 39, 709–717, doi: 10.1007/s10295-011-1077-6 (2012). 22212343

[b4] Choi O., Kim T., Woo H. M. & Um Y. Electricity-driven metabolic shift through direct electron uptake by electroactive heterotroph *Clostridium pasteurianum*. Sci. Rep. 4, 6961, doi: 10.1038/srep06961 (2014). 25376371 PMC4223642

[b5] Dabrock B., Bahl H. & Gottschalk G. Parameters affecting solvent production by *Clostridium pasteurianum*. Appl. Environ. Microbiol. 58, 1233–1239 (1992). 16348691 10.1128/aem.58.4.1233-1239.1992 PMC195580

[b6] Gottschalk G. in Bacterial metabolism (Springer-Verlag, 1986).

[b7] Heyndrickx M., Devos P., Vancanneyt M. & Deley J. The fermentation of glycerol by *Clostridium butyricum* LMG 1212t_2_ and LMG 1213t_1_ and *C. pasteurianum* LMG 3285. Appl. Microbiol. Biotechnol. 34, 637–642 (1991).

[b8] Nakas J. P., Schaedle M., Parkinson C. M., Coonley C. E. & Tanenbaum S. W. System development for linked fermentation production of solvents from algal biomass. Appl. Environ. Microbiol. 46, 1017–1023 (1983). 16346410 10.1128/aem.46.5.1017-1023.1983 PMC239513

[b9] Johnson D. T. & Taconi K. A. The glycerin glut: Options for the value-added conversion of crude glycerol resulting from biodiesel production. Environ. Prog. 26, 338–348, doi: 10.1002/ep.10225 (2007).

[b10] Yazdani S. S. & Gonzalez R. Anaerobic fermentation of glycerol: A path to economic viability for the biofuels industry. Curr. Opin. Biotechnol. 18, 213–219, doi: 10.1016/j.copbio.2007.05.002 (2007). 17532205

[b11] da Silva G. P., Mack M. & Contiero J. Glycerol: A promising and abundant carbon source for industrial microbiology. Biotechnol. Adv. 27, 30–39, doi: 10.1016/j.biotechadv.2008.07.006 (2009). 18775486

[b12] Yang F. X., Hanna M. A. & Sun R. C. Value-added uses for crude glycerol-a byproduct of biodiesel production. Biotechnol. Biofuels 5, 13, doi: 10.1186/1754-6834-5-13 (2012). 22413907 PMC3313861

[b13] Siles López J. Á., Martín Santos M. D. L. Á., Chica Pérez A. F. & Martín Martín A. Anaerobic digestion of glycerol derived from biodiesel manufacturing. Bioresour. Technol. 100, 5609–5615 (2009). 19592236 10.1016/j.biortech.2009.06.017

[b14] Homann T., Tag C., Biebl H., Deckwer W.-D. & Schink B. Fermentation of glycerol to 1,3-propanediol by *Klebsiella* and *Citrobacter* strains. Appl. Microbiol. Biotechnol. 33, 121–126, doi: 10.1007/bf00176511 (1990).

[b15] Shams Yazdani S. & Gonzalez R. Engineering *Escherichia coli* for the efficient conversion of glycerol to ethanol and co-products. Metab. Eng. 10, 340–351 (2008). 18840539 10.1016/j.ymben.2008.08.005

[b16] Lee S. Y. . Fermentative butanol production by clostridia. Biotechnol. Bioeng. 101, 209–228, doi: 10.1002/bit.22003 (2008). 18727018

[b17] Marraffini L. A. & Sontheimer E. J. Self versus non-self discrimination during CRISPR RNA-directed immunity. Nature 463, 568–571 (2010). 20072129 10.1038/nature08703 PMC2813891

[b18] Roberts R. J. Restriction enzymes and their isoschizomers. Nucleic Acids Res. 15, R189–R215 (1987). 3033611 10.1093/nar/15.suppl.r189 PMC339886

[b19] Bolotin A., Quinquis B., Sorokin A. & Ehrlich S. D. Clustered regularly interspaced short palindrome repeats (CRISPRs) have spacers of extrachromosomal origin. Microbiology 151, 2551–2561 (2005). 16079334 10.1099/mic.0.28048-0

[b20] Jansen R., Embden J. D. A. V., Gaastra W. & Schouls L. M. Identification of genes that are associated with DNA repeats in prokaryotes. Mol. Microbiol. 43, 1565–1575 (2002). 11952905 10.1046/j.1365-2958.2002.02839.x

[b21] Roberts R. J., Vincze T., Posfai J. & Macelis D. REBASE—a database for DNA restriction and modification: enzymes, genes and genomes. Nucleic Acids Res. 43, D298–D299 (2015). 25378308 10.1093/nar/gku1046 PMC4383893

[b22] Grissa I., Vergnaud G. & Pourcel C. The CRISPRdb database and tools to display CRISPRs and to generate dictionaries of spacers and repeats. BMC Bioinformatics 8, 172 (2007). 17521438 10.1186/1471-2105-8-172 PMC1892036

[b23] Flusberg B. A. . Direct detection of DNA methylation during single-molecule, real-time sequencing. Nat. Meth. 7, 461–465 (2010). 10.1038/nmeth.1459 PMC2879396 20453866

[b24] Jones D. T. & Woods D. R. Acetone-butanol fermentation revisited. Microbiol. Rev. 50, 484–524 (1986). 3540574 10.1128/mr.50.4.484-524.1986 PMC373084

[b25] Brown S. D. . Comparison of single-molecule sequencing and hybrid approaches for finishing the genome of *Clostridium autoethanogenum* and analysis of CRISPR systems in industrial relevant Clostridia. Biotechnol. Biofuels 7, 40, doi: 10.1186/1754-6834-7-40 (2014). 24655715 PMC4022347

[b26] Zhou Y., Liang Y., Lynch K. H., Dennis J. J. & Wishart D. S. PHAST: A fast phage search tool. Nucleic Acids Res. 39, W347–W352, doi: 10.1093/nar/gkr485 (2011). 21672955 PMC3125810

[b27] Fortier L.-C. & Moineau S. Morphological and genetic diversity of temperate phages in *Clostridium difficile*. Appl. Environ. Microbiol. 73, 7358–7366, doi: 10.1128/aem.00582-07 (2007). 17890338 PMC2168219

[b28] Jensen T. O., Kvist T., Mikkelsen M. J. & Westermann P. Production of 1,3-PDO and butanol by a mutant strain of *Clostridium pasteurianum* with increased tolerance towards crude glycerol. AMB Express 2, 44, doi: 10.1186/2191-0855-2-44 (2012). 22901717 PMC3492062

[b29] Khanna S., Goyal A. & Moholkar V. S. Production of *n*-butanol from biodiesel derived crude glycerol using *Clostridium pasteurianum* immobilized on Amberlite. Fuel 112, 557–561 (2013).

[b30] Venkataramanan K. . Impact of impurities in biodiesel-derived crude glycerol on the fermentation by *Clostridium pasteurianum* ATCC 6013. Appl. Microbiol. Biotechnol. 93, 1325–1335 (2012). 22202963 10.1007/s00253-011-3766-5

[b31] Pyne M. E., Moo-Young M., Chung D. A. & Chou C. P. Development of an electrotransformation protocol for genetic manipulation of *Clostridium pasteurianum*. Biotechnol. Biofuels 6, 50, doi: 10.1186/1754-6834-6-50 (2013). 23570573 PMC3658993

[b32] Pyne M. E., Moo-Young M., Chung D. A. & Chou C. P. Expansion of the genetic toolkit for metabolic engineering of *Clostridium pasteurianum*: chromosomal gene disruption of the endogenous CpaAI restriction enzyme. Biotechnol. biofuels 7, 163, doi: 10.1186/s13068-014-0163-1 (2014). 25431621 PMC4245778

[b33] Sandoval N. R., Venkataramanan K. P., Groth T. S. & Papoutsakis E. T. Whole-genome sequence of an evolved *Clostridium pasteurianum* strain reveals Spo0A deficiency responsible for increased butanol production and superior growth. Biotechnol. biofuels 8, 227, doi: 10.1186/s13068-015-0408-7 (2015). 26705421 PMC4690370

[b34] Pyne M. E., Bruder M., Moo-Young M., Chung D. A. & Chou C. P. Technical guide for genetic advancement of underdeveloped and intractable *Clostridium*. Biotechnol. Adv. 32, 623–641, doi: 10.1016/j.biotechadv.2014.04.003 (2014). 24768687

[b35] Poehlein A., Grosse-Honebrink A., Zhang Y., Minton N. P. & Daniel R. Complete genome sequence of the nitrogen-fixing and solvent-producing *Clostridium pasteurianum* DSM 525. Genome Announc. 3, e01591–01514, doi: 10.1128/genomeA.01591-14 (2015). 25700415 PMC4335339

[b36] Rotta C. . Closed genome sequence of *Clostridium pasteurianum* ATCC 6013. Genome Announc. 3, e01596–01514, doi: 10.1128/genomeA.01596-14 (2015). 25700419 PMC4335343

[b37] Pyne M. E. . Improved draft genome sequence of *Clostridium pasteurianum* strain ATCC 6013 (DSM 525) using a hybrid next-generation sequencing approach. Genome Announc. 2, e00790–00714, doi: 10.1128/genomeA.00790-14 (2014). 25103768 PMC4125779

[b38] Gervasi T., Curto R., Narbad A. & Mayer M. Complete genome sequence of ΦCP51, a temperate bacteriophage of *Clostridium perfringens*. Arch. Virol. 158, 2015–2017, doi: 10.1007/s00705-013-1647-1 (2013). 23575881

[b39] Zimmer M., Scherer S. & Loessner M. J. Genomic analysis of *Clostridium perfringens* bacteriophage φ3626, which integrates into *guaA* and possibly affects sporulation. J. Bacteriol. 184, 4359–4368 (2002). 12142405 10.1128/JB.184.16.4359-4368.2002 PMC135250

[b40] Wiegel J., Tanner R. & Rainey F. A. in The Prokaryotes Vol. 4 (eds Dworkin M. .) 654–678 (Springer, 2006).

[b41] Kolek J., Sedlář K., Provazník I. & Patáková P. Draft genome sequence of *Clostridium pasteurianum* NRRL B-598, a potential butanol or hydrogen producer. Genome Announc. 2, e00192–00114, doi: 10.1128/genomeA.00192-14 (2014). 24652980 PMC3961727

[b42] Rappert S., Song L., Sabra W., Wang W. & Zeng A.-P. Draft genome sequence of type strain *Clostridium pasteurianum* DSM 525 (ATCC 6013), a promising producer of chemicals and fuels. Genome Announc. 1, e00232–00212, doi: 10.1128/genomeA.00232-12 (2013). 23469350 PMC3587944

[b43] Carnahan J. E., Mortenson L. E., Mower H. F. & Castle J. E. Nitrogen fixation in cell-free extracts of *Clostridium pasteurianum*. Biochim. Biophys. Acta 44, 520–535 (1960). 14448610 10.1016/0006-3002(60)91606-1

[b44] Mortenson L. E. Ferredoxin and ATP, requirements for nitrogen fixation in cell-free extracts of *Clostridium pasteurianum*. Proc. Natl. Acad. Sci. USA 52, 272–279 (1964). 14206590 10.1073/pnas.52.2.272 PMC300271

[b45] Wang S. Z., Chen J. S. & Johnson J. L. Distinct structural features of the alpha and beta subunits of nitrogenase molybdenum-iron protein of *Clostridium pasteurianum*: an analysis of amino acid sequences. Biochemistry 27, 2800–2810, doi: 10.1021/bi00408a021 (1988). 2840948

[b46] Chen J.-S., Wang S.-Z. & Johnson J. in Nitrogen Fixation (eds Gresshoff PeterM., Roth L. Evans, Stacey Gary, Newton WilliamE.) Ch. 48, 483–490 (Springer: US, , 1990).

[b47] Brigle K. E., Newton W. E. & Dean D. R. Complete nucleotide sequence of the *Azotobacter vinelandii* nitrogenase structural gene cluster. Gene 37, 37–44 (1985). 3863780 10.1016/0378-1119(85)90255-0

[b48] Johnson J., Wang S.-Z. & Chen J.-S. In Genetics and, olecular Biology of Anaerobic Bacteria Brock/Springer Series in Contemporary Bioscience (ed Sebald Madaleine) Ch. 26, 373–381 (Springer: New York, , 1993).

[b49] Wang S.-Z., Chen J.-S. & Johnson J. L. The presence of five *nifH*-like sequences in *Clostridium pasteurianum*: sequence divergence and transcription properties. Nucleic Acids Res. 16, 439–454 (1988). 2829127 10.1093/nar/16.2.439 PMC334671

[b50] Zinoni F., Robson R. M. & Robson R. L. Organization of potential alternative nitrogenase genes from *Clostridium pasteurianum*. BBA - Gene Struct. Expr. 1174, 83–86 (1993). 10.1016/0167-4781(93)90096-v 8334167

[b51] Dilworth M. J., Eady R. R., Robson R. L. & Miller R. W. Ethane formation from acetylene as a potential test for vanadium nitrogenase *in vivo*. Nature 327, 167–168 (1987).

[b52] Richards D. F., Linnett P. E., Oultram J. D. & Young M. Restriction endonucleases in *Clostridium pasteurianum* ATCC 6013 and *C. thermohydrosulfuricum* DSM 568. J. Gen. Microbiol. 134, 3151–3157 (1988). 3269388 10.1099/00221287-134-12-3151

[b53] Raleigh E. A. & Wilson G. *Escherichia coli* K-12 restricts DNA containing 5-methylcytosine. Proc. Natl. Acad. Sci. USA 83, 9070–9074, doi: 10.1073/pnas.83.23.9070 (1986). 3024165 PMC387076

[b54] Makarova K. S. . Evolution and classification of the CRISPR–Cas systems. Nat. Rev. Micro. 9, 467–477 (2011). 10.1038/nrmicro2577 PMC3380444 21552286

[b55] Grissa I., Vergnaud G. & Pourcel C. CRISPRFinder: a web tool to identify clustered regularly interspaced short palindromic repeats. Nucleic Acids Res. 35, W52–W57, doi: 10.1093/nar/gkm360 (2007). 17537822 PMC1933234

[b56] Deltcheva E. . CRISPR RNA maturation by trans-encoded small RNA and host factor RNase III. Nature 471, 602–607, doi: 10.1038/nature09886 (2011). 21455174 PMC3070239

[b57] Carte J., Wang R., Li H., Terns R. M. & Terns M. P. Cas6 is an endoribonuclease that generates guide RNAs for invader defense in prokaryotes. Genes Dev. 22, 3489–3496 (2008). 19141480 10.1101/gad.1742908 PMC2607076

[b58] Haurwitz R. E., Jinek M., Wiedenheft B., Zhou K. & Doudna J. A. Sequence-and structure-specific RNA processing by a CRISPR endonuclease. Science 329, 1355–1358 (2010). 20829488 10.1126/science.1192272 PMC3133607

[b59] Chylinski K., Le Rhun A. & Charpentier E. The tracrRNA and Cas9 families of type II CRISPR-Cas immunity systems. RNA Biol. 10, 726–737 (2013). 23563642 10.4161/rna.24321 PMC3737331

[b60] Sinkunas T. . Cas3 is a single‐stranded DNA nuclease and ATP‐dependent helicase in the CRISPR/Cas immune system. EMBO J. 30, 1335–1342 (2011). 21343909 10.1038/emboj.2011.41 PMC3094125

[b61] Haft D. H., Selengut J., Mongodin E. F. & Nelson K. E. A guild of 45 CRISPR-associated (Cas) protein families and multiple CRISPR/Cas subtypes exist in prokaryotic genomes. PLoS Comput. Biol. 1, e60, doi: 10.1371/journal.pcbi.0010060 (2005). 16292354 PMC1282333

[b62] Richter H. . Characterization of CRISPR RNA processing in *Clostridium thermocellum* and *Methanococcus maripaludis*. Nucleic Acids Res. 40, 9887–9896 (2012). 22879377 10.1093/nar/gks737 PMC3479195

[b63] Zuker M. Mfold web server for nucleic acid folding and hybridization prediction. Nucleic Acids Res. 31, 3406–3415 (2003). 12824337 10.1093/nar/gkg595 PMC169194

[b64] Kunin V., Sorek R. & Hugenholtz P. Evolutionary conservation of sequence and secondary structures in CRISPR repeats. Genome Biol. 8, R61, doi: 10.1186/gb-2007-8-4-r61 (2007). 17442114 PMC1896005

[b65] Shah S. A., Erdmann S., Mojica F. J. & Garrett R. A. Protospacer recognition motifs: mixed identities and functional diversity. RNA Biol. 10, 891–899 (2013). 23403393 10.4161/rna.23764 PMC3737346

[b66] Gheshlaghi R., Scharer J. M., Moo-Young M. & Chou C. P. Metabolic pathways of clostridia for producing butanol. Biotechnol. Adv. 27, 764–781, doi: 10.1016/j.biotechadv.2009.06.002 (2009). 19539744

[b67] Kopke M. . *Clostridium ljungdahlii* represents a microbial production platform based on syngas. Proc. Natl. Acad. Sci. USA 107, 13087–13092 (2010). 20616070 10.1073/pnas.1004716107 PMC2919952

[b68] Bender R., Andreesen J. R. & Gottschalk G. 2-Keto-3-deoxygluconate, an intermediate in the fermentation of gluconate by clostridia. J. Bacteriol. 107, 570–573 (1971). 5113600 10.1128/jb.107.2.570-573.1971 PMC246963

[b69] Nolling J. . Genome sequence and comparative analysis of the solvent-producing bacterium *Clostridium acetobutylicum*. J. Bacteriol. 183, 4823–4838 (2001). 11466286 10.1128/JB.183.16.4823-4838.2001 PMC99537

[b70] Gottschalk G. & Barker H. Presence and stereospecificity of citrate synthase in anaerobic bacteria. Biochemistry 6, 1027–1034 (1967). 6032450 10.1021/bi00856a011

[b71] Biebl H., Menzel K., Zeng A.-P. & Deckwer W.-D. Microbial production of 1,3-propanediol. Appl. Microbiol. Biotechnol. 52, 289–297 (1999). 10531640 10.1007/s002530051523

[b72] Luers F., Seyfried M., Daniel R. & Gottschalk G. Glycerol conversion to 1,3-propanediol by *Clostridium pasteurianum*: cloning and expression of the gene encoding 1,3-propanediol dehydrogenase. FEMS Microbiol. Lett. 154, 337–345 (1997). 9311132 10.1111/j.1574-6968.1997.tb12665.x

[b73] Sun J., van den Heuvel J., Soucaille P., Qu Y. & Zeng A. P. Comparative genomic analysis of *dha* regulon and related genes for anaerobic glycerol metabolism in bacteria. Biotechnol. Prog. 19, 263–272 (2003). 12675558 10.1021/bp025739m

[b74] Macis L., Daniel R. & Gottschalk G. Properties and sequence of the coenzyme B-12-dependent glycerol dehydratase of *Clostridium pasteurianum*. FEMS Microbiol. Lett. 164, 21–28 (1998). 9675846 10.1111/j.1574-6968.1998.tb13062.x

[b75] Daniel R., Bobik T. A. & Gottschalk G. Biochemistry of coenzyme B12-dependent glycerol and diol dehydratases and organization of the encoding genes. FEMS Microbiol. Rev. 22, 553–566 (1998). 9990728 10.1111/j.1574-6976.1998.tb00387.x

[b76] Johnson E. E. & Rehmann L. The role of 1,3-propanediol production in fermentation of glycerol by *Clostridium pasteurianum*. Bioresour. Technol. 209, 1–7 (2016). 26946434 10.1016/j.biortech.2016.02.088

[b77] Gonzalez R., Murarka A., Dharmadi Y. & Yazdani S. S. A new model for the anaerobic fermentation of glycerol in enteric bacteria: Trunk and auxiliary pathways in *Escherichia coli*. Metab. Eng. 10, 234–245 (2008). 18632294 10.1016/j.ymben.2008.05.001

[b78] Li Y. C. . Combined inactivation of the *Clostridium cellulolyticum* lactate and malate dehydrogenase genes substantially increases ethanol yield from cellulose and switchgrass fermentations. Biotechnol. Biofuels 5, 2, doi: 10.1186/1754-6834-5-2 (2012). 22214220 PMC3268733

[b79] Mortenson L., Valentine R. & Carnahan J. E. Ferredoxin in the phosphoroclastic reaction of pyruvic acid and its relation to nitrogen fixation in *Clostridium pasteurianum*. J. Biol. Chem. 238, 794–800 (1963).

[b80] Weidner G. & Sawers G. Molecular characterization of the genes encoding pyruvate formate-lyase and its activating enzyme of *Clostridium pasteurianum*. J. Bacteriol. 178, 2440–2444 (1996). 8636053 10.1128/jb.178.8.2440-2444.1996 PMC177960

[b81] Thauer R. K., Kirchniawy F. H. & Jungermann K. A. Properties and function of the pyruvate‐formate‐lyase reaction in Clostridiae. Eur. J. Biochem. 27, 282–290 (1972). 4340563 10.1111/j.1432-1033.1972.tb01837.x

[b82] Mortenson L. E., Valentine R. C. & Carnahan J. E. An electron transport factor from *Clostridium pasteurianum*. Biochem. Biophys. Res. Commun. 7, 448–452 (1962). 14476372 10.1016/0006-291x(62)90333-9

[b83] Meyer J. & Gagnon J. Primary structure of hydrogenase from *Clostridium pasteurianum*. Biochemistry 30, 9697–9704 (1991). 1911757 10.1021/bi00104a018

[b84] Chen J.-S. & Blanchard D. K. Purification and properties of the H_2_-oxidizing (uptake) hydrogenase of the N_2_-fixing anaerobe *Clostridium pasteurianum* W5. Biochem. Biophys. Res. Commun. 122, 9–16 (1984). 6331453 10.1016/0006-291x(84)90431-5

[b85] Boynton Z. L., Bennett G. N. & Rudolph F. B. Cloning, sequencing, and expression of genes encoding phosphotransacetylase and acetate kinase from *Clostridium acetobutylicum* ATCC 824. Appl. Environ. Microbiol. 62, 2758–2766 (1996). 8702268 10.1128/aem.62.8.2758-2766.1996 PMC168061

[b86] Cooksley C. M. . Targeted mutagenesis of the *Clostridium acetobutylicum* acetone-butanol-ethanol fermentation pathway. Metab. Eng. 14, 630–641, doi: 10.1016/j.ymben.2012.09.001 (2012). 22982601

[b87] Fontaine L. . Molecular characterization and transcriptional analysis of *adhE2*, the gene encoding the NADH-dependent aldehyde/alcohol dehydrogenase responsible for butanol production in alcohologenic cultures of *Clostridium acetobutylicum* ATCC 824. J. Bacteriol. 184, 821–830 (2002). 11790753 10.1128/JB.184.3.821-830.2002 PMC139506

[b88] Nair R. V., Bennett G. N. & Papoutsakis E. T. Molecular characterization of an aldehyde/alcohol dehydrogenase gene from *Clostridium acetobutylicum* ATCC 824. J. Bacteriol. 176, 871–885 (1994). 8300540 10.1128/jb.176.3.871-885.1994 PMC205125

[b89] Meng Y. & Li J. L. Cloning, expression and characterization of a thiolase gene from *Clostridium pasteurianum*. Biotechnol. Lett. 28, 1227–1232 (2006). 16802096 10.1007/s10529-006-9089-4

[b90] Bennett G. N. & Rudolph F. B. The central metabolic pathway from acetyl‐CoA to butyryl‐CoA in *Clostridium acetobutylicum*. FEMS Microbiol. Rev. 17, 241–249 (1995).

[b91] Bruant G., Lévesque M.-J., Peter C., Guiot S. R. & Masson L. Genomic analysis of carbon monoxide utilization and butanol production by *Clostridium carboxidivorans* strain P7T. Plos One 5, 1–12 (2010). 10.1371/journal.pone.0013033 PMC2946384 20885952

[b92] Wietzke M. & Bahl H. The redox-sensing protein Rex, a transcriptional regulator of solventogenesis in *Clostridium acetobutylicum*. Appl. Microbiol. Biotechnol. 96, 749–761 (2012). 22576944 10.1007/s00253-012-4112-2

[b93] Jungermann K., Thauer R. K., Leimenstoll G. & Decker K. Function of reduced pyridine nucleotide-ferredoxin oxidoreductases in saccharolytic Clostridia. BBA - Bioenergetics 305, 268–280 (1973). 4147457 10.1016/0005-2728(73)90175-8

[b94] Jungermann K., Leimenstoll G., Rupprecht E. & Thauer R. K. Demonstration of NADH-ferredoxin reductase in two saccharolytic Clostridia. Arch. Microbiol. 80, 370–372 (1971). 10.1007/BF00406223 4332095

[b95] Petitdemange H., Cherrier C., Raval G. & Gay R. Regulation of the NADH and NADPH-ferredoxin oxidoreductases in Clostridia of the butyric group. BBA – Gen. Subjects 421, 334–347 (1976). 10.1016/0304-4165(76)90300-7 3218

[b96] Rao G. & Mutharasan R. Altered electron flow in continuous cultures of *Clostridium acetobutylicum* induced by viologen dyes. Appl. Environ. Microbiol. 53, 1232–1235 (1987). 16347357 10.1128/aem.53.6.1232-1235.1987 PMC203846

[b97] Herrmann G., Jayamani E., Mai G. & Buckel W. Energy conservation via electron-transferring flavoprotein in anaerobic bacteria. J. Bacteriol. 190, 784–791 (2008). 18039764 10.1128/JB.01422-07 PMC2223574

[b98] Li F. . Coupled ferredoxin and crotonyl coenzyme A (CoA) reduction with NADH catalyzed by the butyryl-CoA dehydrogenase/Etf complex from *Clostridium kluyveri*. J. Bacteriol. 190, 843–850 (2008). 17993531 10.1128/JB.01417-07 PMC2223550

[b99] Winzer T. . Morphinan biosynthesis in opium poppy requires a P450-oxidoreductase fusion protein. Science 349, 309–312 (2015). 26113639 10.1126/science.aab1852

[b100] Fischer R. J., Helms J. & Dürre P. Cloning, sequencing, and molecular analysis of the *sol* operon of *Clostridium acetobutylicum*, a chromosomal locus involved in solventogenesis. J. Bacteriol. 175, 6959–6969 (1993). 8226639 10.1128/jb.175.21.6959-6969.1993 PMC206823

[b101] Harris J., Mulder R., Kell D. B., Walter R. P. & Morris J. G. Solvent production by *Clostridium pasteurianum* in media of high sugar content. Biotechnol. Lett. 8, 889–892 (1986).

[b102] Maddox I. . The cause of “acid crash” and “acidogenic fermentations” during the batch acetone-butanol-ethanol (ABE) fermentation process. J. Mol. Microbiol. Biotechnol. 2, 95–100 (2000). 10937493

[b103] Moon C. & Lee C. H., Sang, B. I. & Um, Y. Optimization of medium compositions favoring butanol and 1,3-propanediol production from glycerol by *Clostridium pasteurianum*. Bioresour. Technol. 102, 10561–10568, doi: 10.1016/j.biortech.2011.08.094 (2011). 21945663

[b104] Girbal L., Croux C., Vasconcelos I. & Soucaille P. Regulation of metabolic shifts in *Clostridium acetobutylicum* ATCC 824. FEMS Microbiol. Rev. 17, 287–297 (1995).

[b105] Huang K., Huang S., Rudolph F. B. & Bennett G. N. Identification and characterization of a second butyrate kinase from *Clostridium acetobutylicum* ATCC 824. J. Mol. Microbiol. Biotechnol. 2, 33–38 (2000). 10937485

[b106] Walter K. A., Bennett G. & Papoutsakis E. T. Molecular characterization of two *Clostridium acetobutylicum* ATCC 824 butanol dehydrogenase isozyme genes. J. Bacteriol. 174, 7149–7158 (1992). 1385386 10.1128/jb.174.22.7149-7158.1992 PMC207405

[b107] Sauer U. & Durre P. Differential induction of genes related to solvent formation during the shift from acidogenesis to solventogenesis in continuous culture of *Clostridium acetobutylicum*. FEMS Microbiol. Lett. 125, 115–120 (1995).

[b108] Rainey F. A., Hollen B. J. & Small A. in Bergey’s Manual of Systematic Bacteriology Vol. 3 The Firmicutes (eds Vos P. De . ) 738–830 (Springer, 2009).

[b109] Booth I. & Morris J. Carbohydrate transport in *Clostridium pasteurianum*. Biosci. Rep. 2, 47–53 (1982). 6277409 10.1007/BF01142198

[b110] Mitchell W. J., Roohi M. S., Mosely M. J. & Booth I. R. Regulation of carbohydrate utilization in *Clostridium pasteurianum*. J. Gen. Microbiol. 133, 31–36 (1987).

[b111] Booth I. & Morris J. Proton-motive force in the obligately anaerobic bacterium *Clostridium pasteurianum*: a role in galactose and gluconate uptake. FEBS Lett. 59, 153–157 (1975). 6313 10.1016/0014-5793(75)80364-4

[b112] Heyndrickx M., Devos P. & Deley J. Fermentation characteristics of *Clostridium pasteurianum* LMG 3285 grown on glucose and mannitol. J. Appl. Bacteriol. 70, 52–58 (1991).

[b113] San K.-Y. . Metabolic engineering through cofactor manipulation and its effects on metabolic flux redistribution in *Escherichia coli*. Metab. Eng. 4, 182–192 (2002). 12009797 10.1006/mben.2001.0220

[b114] Bender R. & Gottschalk G. Purification and properties of d-gluconate dehydratase from *Clostridium pasteurianum*. Eur. J. Biochem. 40, 309–321 (1973). 4772682 10.1111/j.1432-1033.1973.tb03198.x

[b115] VanBriesen J. M. Evaluation of methods to predict bacterialyield using thermodynamics. Biodegradation 13, 171–190 (2002). 12498215 10.1023/a:1020887214879

[b116] Roohi M. S. & Mitchell W. J. Regulation of sorbitol metabolism by glucose in *Clostridium pasteurianum*: a role for inducer exclusion. J. Gen. Microbiol. 133, 2207–2215 (1987).

[b117] Sarchami T., Johnson E. & Rehmann L. Optimization of fermentation condition favoring butanol production from glycerol by *Clostridium pasteurianum* DSM 525. Bioresour. Technol. 208, 73–80 (2016). 26922315 10.1016/j.biortech.2016.02.062

[b118] Malaviya A., Jang Y. S. & Lee S. Y. Continuous butanol production with reduced byproducts formation from glycerol by a hyper producing mutant of *Clostridium pasteurianum*. Appl. Microbiol. Biotechnol. 93, 1485–1494, doi: 10.1007/s00253-011-3629-0 (2012). 22052388

[b119] Kim B. H., Bellows P., Datta R. & Zeikus J. G. Control of carbon and electron flow in *Clostridium acetobutylicum* fermentations - Utilization of carbon monoxide to inhibit hydrogen production and to enhance butanol yields. Appl. Environ. Microbiol. 48, 764–770 (1984). 16346643 10.1128/aem.48.4.764-770.1984 PMC241610

[b120] Grant J. R. & Stothard P. The CGView Server: a comparative genomics tool for circular genomes. Nucleic Acids Res. 36, W181–W184 (2008). 18411202 10.1093/nar/gkn179 PMC2447734

