# Supplementary material for: Genome-directed analysis of prophage excision, host defence systems, and central fermentative metabolism in Clostridium pasteurianum
Source: Sci Rep. 2016 Sep 19;6:26228. doi: 10.1038/srep26228 (PMC5027557; doi:10.1038/srep26228)
Supplement: Supplementary Information [file srep26228-s1.pdf]

Genome-directed analysis of prophage excision, host defence systems, and central fermentative metabolism in *Clostridium pasteurianum*

Michael E. Pyne[1,*,†], Xuejia Liu[1], Murray Moo-Young[1], Duane A. Chung[1,2,3,*], C. Perry Chou[1,*]

Department of Chemical Engineering, University of Waterloo, Waterloo, Ontario, Canada[1]; Department of Pathology and Molecular Medicine, McMaster University, Ontario, Canada[2]; Algaeneers Inc. and Neemo Inc., Hamilton, Ontario, Canada[3];

*Address correspondence to Michael E. Pyne, michael.pyne@concordia.ca; Duane A. Chung, duane.chung@uwaterloo.ca; and C. Perry Chou, cpchou@uwaterloo.ca

†Present address: Department of Biology and Centre for Structural and Functional Genomics, Concordia University, Montréal, Québec, Canada.

Supplementary Figure S1

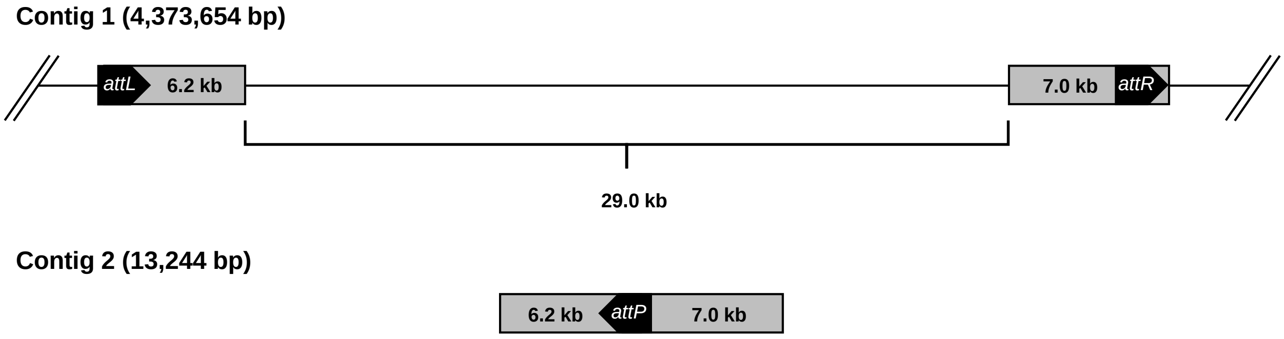

Supplementary Figure S1 – Contig size and arrangement following next-generation genome sequencing of *C. pasteurianum*. Two distinct chromosomal regions (6.2 kb and 7.0 kb) could be identified within the disproportionately-sized contigs 1 and 2 following SMRT sequencing and assembly. These regions possess 28 bp of overlap within contig 2 (*attP*), while the overlap sequence was found to be preserved on the ends of the regions within contig 1 (*attL* and *attR*). Chromosomal regions are not depicted to scale.

Supplementary Figure S2

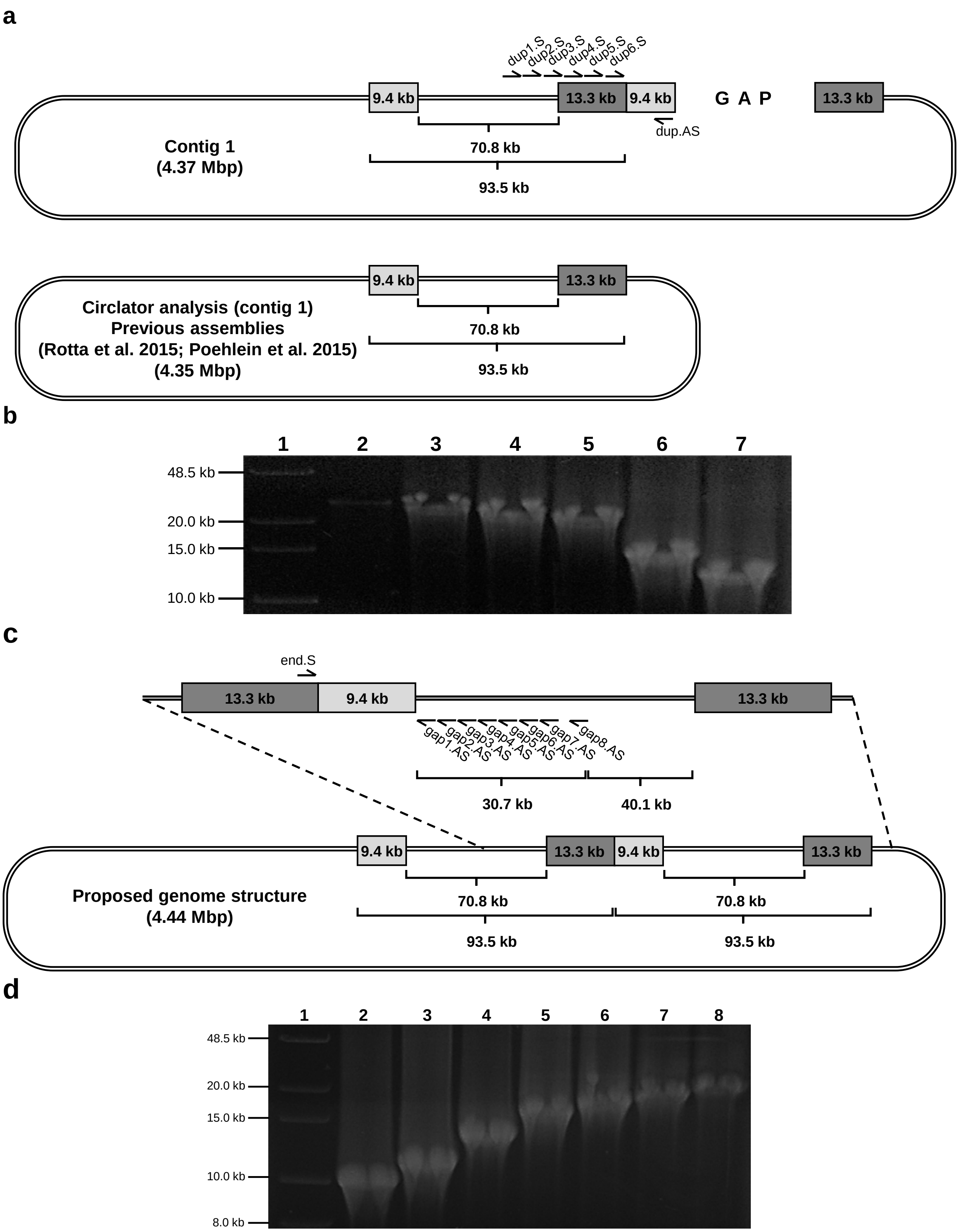

Supplementary Figure S2 – Comparison of the *C. pasteurianum* contig presented in this study with previous assemblies and strategies for genome closing. **(a)** Structure, sizes, and arrangement of key genomic regions within contig 1 and previous *C. pasteurianum* genome sequences. Circlator analysis of contig 1 predicted genome closing through exclusion of the 9.4 kb duplication (light shading) at the 3' end of contig 1 and circularization through overlapping 13.3 kb (dark shading) regions. The resulting closed genome is analogous to the two existing closed *C. pasteurianum* genome assemblies[1,2]. Primer binding sites are shown for PCR verification. Genomic regions and primers are not depicted to scale. **(b)** PCR verification of the genomic structure and arrangement of key regions at the 3' end of contig 1. Orientation and approximate arrangement of relevant genomic regions and PCR primers are depicted in Supplementary Fig. S2a online. Lane 1: marker; lane 2: 27,041 bp product (dup1.S + dup.AS); lane 3: 23,669 bp product (dup2.S + dup.AS); lane 4: 21,151 bp product (dup3.S + dup.AS); lane 5: 20,324 bp product (dup4.S + dup.AS); lane 6: 13,977 bp product (dup5.S + dup.AS); lane 7: 11,903 bp product (dup6.S + dup.AS). **(c)** Proposed *C. pasteurianum* genome closing hypothesis depicting a 93.5 kb chromosomal duplication. The contig 1 gap depicted in Supplementary Fig. S2a could be closed by proposing a 93.5 kb chromosomal duplication. An enlarged genomic region is depicted above the closed genome assembly and PCR primer binding sites employed for probing our hypothesis are shown (see Supplementary Fig. S2d online). The 30.7 kb region corresponds to an extension into the genome gap achieved using PCR based on our proposed duplication hypothesis. Genomic regions and primers are not depicted to scale. **(d)** Probing our proposed genome closing hypothesis using long range PCR. A sample agarose gel of PCR products from one gap extension experiment is shown. Orientation and approximate arrangement of relevant genomic regions and PCR primers are depicted in Supplementary Fig. S2c online. Lane 1:

marker; lane 2: 10,983 bp product (end.S + gap1.AS); lane 3: 11,958 bp product (end.S + gap2.AS); lane 4: 14,429 bp product (end.S + gap3.AS); lane 5: 16,939 bp product (end.S + gap4.AS); lane 6: 18,654 bp product (end.S + gap5.AS); lane 7: 20,494 bp product (end.S + gap6.AS); lane 8: 22,708 bp product (end.S + gap7.AS).

Supplementary Table S1. Genes and functional assignments encoded by phage φ6013

ORF	Prophage locus tag	Genomic (prophage) start coordinates	Genomic (prophage) stop coordinates	Functional assignment
1	CP6013_3170	3471803	3472324	Terminase, small subunit
2	CP6013_3171	3472311	3473708	Terminase, large subunit
3	CP6013_3172	3473711	3475216	Minor capsid protein
4	CP6013_3173	3475219	3475686	Minor capsid protein
5	CP6013_3174	3475683	3476852	Minor capsid protein
6	CP6013_3175	3476852	3477175	
7	CP6013_3176	3477243	3477485	
8	CP6013_3177	3477496	3477696	
9	CP6013_3178	3477679	3477843	
10	CP6013_3179	3477982	3478581	Minor structural protein
11	CP6013_3180	3478597	3479562	Capsid protein
12	CP6013_3181	3479573	3479860	
13	CP6013_3182	3479863	3480222	
14	CP6013_3183	3480231	3480557	
15	CP6013_3184	3480557	3480940	
16	CP6013_3185	3480940	3481326	
17	CP6013_3186	3481337	3481792	
18	CP6013_3187	3481814	3482143	
19	CP6013_3188	3482233	3482466	
20	CP6013_3189	3482527	3482946	
21	CP6013_3190	3483011	3487114	Tail tape measure protein
22	CP6013_3191	3487125	3487472	
23	CP6013_3192	3487652	3487921	
24	CP6013_3193	3487987	3488364	
25	CP6013_3194	3488391	3495293	Minor structural protein
26	CP6013_3195	3495336	3495725	Holin
27	CP6013_3196	3495744	3496727	Autolysin
28	CP6013_3197	3496782	3497279	
29	CP6013_3146	3455566	3456615	Integrase
30	CP6013_3147	3456707	3457168	
31	CP6013_3148	3457202	3457642	Repressor
32	CP6013_3149	3457829	3458077	Repressor
33	CP6013_3150	3458094	3458261	Regulator
34	CP6013_3151	3458427	3458567	
35	CP6013_3152	3458601	3458843	
36	CP6013_3153	3458849	3458971	
37	CP6013_3154	3458974	3459207	SpoIIID regulator
38	CP6013_3155	3459226	3459837	Sigma factor (σ^E)
39	CP6013_3156	3459905	3460093	
40	CP6013_3157	3460157	3460618	

41	CP6013_3158	3460632	3460892	
42	CP6013_3159	3460928	3461377	
43	CP6013_3160	3461379	3462542	
44	CP6013_3161	3462664	3463224	
45	CP6013_3162	3463226	3465187	DNA polymerase
46	CP6013_3163	3465202	3466227	
47	CP6013_3164	3466262	3466387	
48	CP6013_3165	3466507	3466653	
49	CP6013_3166	3466688	3469114	Virulence protein
50	CP6013_3167	3469386	3469664	Nuclease
51	CP6013_3168	3469661	3471025	Helicase
52	CP6013_3169	3471038	3471586	

Supplementary Table S2. Comparison of the *C. pasteurianum* genome to closely-related clostridia with completed genome sequences

General features	*C. pasteurianum* ATCC 6013 (DSM 525)	*C. acetobutylicum* ATCC 824	*C. botulinum* Hall A	*C. autoethanogenum* DSM 10061	*C. ljungdahlii* DSM 13528
**Size (Mbp)**	4.4	4.1	3.8	4.4	4.6
**GC content (%)**	29.9	30.9	28.2	31.1	31.1
**Plasmid**	ND	pSOL1 megaplasmid	pBOT3502	ND	ND
**Protein-coding genes**	3,803	3,778	3,332	3,741	4,081
**Average size of protein-coding genes (bp)**	918	930	903	933	942
**Coding density (%)[a]**	79.0	85.0	80.0	80.2	83.0
**tRNA genes**	81	73	81	67	70
**rRNA genes**	30	33	24	27	27
**Reference**	This study	[3]	[4]	[5]	[6]

[a] Calculated by multiplying the average protein-coding gene size by the total number of genes and dividing by the total genome size.

ND: not detected.

Supplementary Table S3. Oligonucleotides employed in this study

Oligonucleotide	Sequence (5’-3’)*
attLB.S	GCAAAGACATCAGGTTTTCAAAAGGGTG
attL.AS	CAACACTGAACACTTATTCACACCTGTACCC
attRP.S	GTTACTACACTTACACAGGAAAATGCAGCTC
attRB.AS	CGTGAAAGAAGATATGGTCAATTCAGAAGAGG
attP.AS	CATTCAATGTCCTCCTTTCCTAATTCCTCAT
Φ6013.S	GGCTGAATCAATAGTTGAGACTTTGGAAGG
Φ6013.AS	CCTTCCAAAGTCTCAACTATTGATTCAGCC
dup1.S	GCAGAGATAATGGAAGAACACCAATGC
dup2.S	GCATAGTCTGATTATAGTAGCAGAAGGAATTGG
dup3.S	GGTGGATTACTGGACAAAGGAAGTTTTTCAAGG
dup4.S	GGTAGTTTCCATTTGCCGCTGATAATGTTTAGTG
dup5.S	GCGAGATTACTGGGTGGATTACAGGAACG
dup6.S	GGCATAAACAATACTGCTTTCAAGTTCC
dup.AS	GGTGCCGTAAACATCAAATAGTACAAGC
end.S	GCATCTGTAGGAAAATCATCTGCAAAAGTTTGTTGAGC
gap1.AS	GCACTGACTGAGGATTGGTTTAGTCC
gap2.AS	GTCAGATTGAATGGCTAAAGGATGCTGG
gap3.AS	GAAAAGCGAAGATAAACCTATACCAAGCC
gap4.AS	GTTTCATAATGTTCATAACCTGCATCTGTGG
gap5.AS	GCTCATCAGGTTCAATATCTACTATTTCCACC
gap6.AS	GCATTATCCAGCAGATTTATGATAACCTGTTC
gap7.AS	CCATGGCTATTATCAAAAGAGGGACTGC
gap8.AS	CCAAGGTAACAATAATACCAAGTACGAAAGG

Supplementary Note

Attempted closing of the *C. pasteurianum* contig gap using long range PCR

Analysis of the 5' and 3' ends of contig 1 revealed 13,342 bp and 9,353 bp sequences that were found elsewhere in the genome separated by 70,816 bp (see Supplementary Fig. S2 online). This finding led us to postulate that the gap region within contig 1 comprises a large 93,551 bp chromosomal duplication, of which 13,342 bp and 9,353 bp, totaling 22,695 bp, was captured within the ends of contig 1. Assuming this duplication hypothesis, the two 93,551 bp regions are adjacent within the chromosome. Surprisingly, only one copy of the 93,551 bp region was found within previous *C. pasteurianum* genome sequences[1,2]. Furthermore, analysis of our genome assembly using Circlator[7] omitted the 9,353 bp sequence at the 3' end of contig 1 and circularized the genome through the resulting overlapping 13,342 bp end regions, yielding a closed genome consistent with previous assemblies[1,2] (see Supplementary Fig. S2 online). Despite this prediction, we successfully amplified several PCR products spanning 27 kb at the end of contig 1 (see Supplementary Fig. S2 online), thus confirming presence of the 9,353 bp region at the 3' end of the contig and validating our distinct genome assembly. The primer sets employed in Supplementary Fig. S2b are not expected to generate PCR products based on previously reported *C. pasteurianum* genome sequences[1,2]. To further probe our genome duplication hypothesis, we employed long range PCR in an attempt to fill in the gap of contig 1, assuming that the gap region corresponds to the 93,551 bp duplicated sequence (see Supplementary Fig. S2 online). By utilizing one unique primer outside of the duplicated region and one primer specific to the duplication, we generated several PCR products consistent with our 93,551 bp genome duplication hypothesis. Agarose gel electrophoresis results from one such PCR gap closing experiment is shown in Supplementary Fig. S2 online. Following extensive

long range PCR analysis, we were successful in extending contig 1 by 30,666 bp (primers end.S + gap8.AS; see Supplementary Fig. S2 online). Due to size limitations associated with PCR, however, we were unable to generate products enclosing the expected remaining 40,150 bp of the contig gap in order to unequivocally confirm the existence of a 93,551 bp chromosomal duplication and close the genome.

References

1 Poehlein, A., Grosse-Honebrink, A., Zhang, Y., Minton, N. P. & Daniel, R. Complete genome sequence of the nitrogen-fixing and solvent-producing *Clostridium pasteurianum* DSM 525. *Genome Announcements* **3**, e01591-01514; doi:10.1128/genomeA.01591-14 (2015).

2 Rotta, C. *et al.* Closed genome sequence of *Clostridium pasteurianum* ATCC 6013. *Genome Announcements* **3**, e01596-01514; doi:10.1128/genomeA.01596-14 (2015).

3 Nolling, J. *et al.* Genome sequence and comparative analysis of the solvent-producing bacterium *Clostridium acetobutylicum*. *J. Bacteriol.* **183**, 4823-4838 (2001).

4 Sebaihia, M. *et al.* Genome sequence of a proteolytic (Group I) *Clostridium botulinum* strain Hall A and comparative analysis of the clostridial genomes. *Genome Res.* **17**, 1082-1092 (2007).

5 Brown, S. D. *et al.* Comparison of single-molecule sequencing and hybrid approaches for finishing the genome of *Clostridium autoethanogenum* and analysis of CRISPR systems in industrial relevant Clostridia. *Biotechnol. Biofuels* **7**, 40; doi:10.1186/1754-6834-7-40 (2014).

6 Kopke, M. *et al.* *Clostridium ljungdahlii* represents a microbial production platform based on syngas. *Proc. Natl. Acad. Sci. U. S. A.* **107**, 13087-13092 (2010).

7 Hunt, M. *et al.* Circlator: automated circularization of genome assemblies using long sequencing reads. *bioRxiv* **16**, 294; doi:10.1186/s13059-015-0849-0 (2015).
